# Beyond will: the empowerment conditions needed to abandon female genital mutilation in Conakry (Guinea), a focused ethnography

**DOI:** 10.1186/s12978-020-00910-1

**Published:** 2020-05-06

**Authors:** Marie-Hélène Doucet, Alexandre Delamou, Hawa Manet, Danielle Groleau

**Affiliations:** 1 McGill University, Division of Social and Transcultural Psychiatry, 1033, Des Pins West, Montreal, Quebec H3A 1A1 Canada; 2 Centre national de formation et de recherche en santé rurale de Mafèrinyah, B.P. 2649, Conakry, Republic of Guinea; 3 Université Gamal Abdel Nasser de Conakry, Conakry, Republic of Guinea; 4 Jewish General Hospital, Lady Davis Institute, 4333 Côte St-Catherine Road, Montreal, Quebec H3T 1E4 Canada

**Keywords:** Qualitative research, Focused ethnography, Positive deviance, Guinea/Conakry, Female genital mutilation (FGM), Social capital, Economic capital, Individualization process, Recherche qualitative, Ethnographie focalisée, Déviance positive, Guinée/Conakry, Mutilations génitales féminines (MGF), Capital social, Capital économique, Processus d’individualisation

## Abstract

**Background:**

Female genital mutilation (FGM) can give rise to immediate and long-term health problems for girls/women. Numerous studies have identified the sociocultural determinants of this tradition, but so far, in a national context where FGM is highly practiced, virtually none have focused on people refusing to have their daughters cut. We therefore aimed to understand the sociocultural dynamics underlying the non-practice of FGM in Guinea, a country which has one of the most prevalent rates of this practice in the world. This research explored the demographic and sociocultural profiles of Guineans who do not practice FGM, as well as their non-practice experience in a context of high FGM prevalence and social pressure.

**Methods:**

We used a “focused ethnography” methodology and conducted semi-structured individual interviews with 30 women and men from different generations (young adults, parents, grandparents) living in Conakry, Guinea.

**Results:**

We found that participants 1) do not disclose their non-practicing status in the same way, and 2) have different experiences with social pressure. A typology was created to describe participants as per their various profiles and experiences, which we named as: 1) the “activists”, 2) the “discrete”, 3) the “courageous”, 4) the “strategists”.

**Discussion:**

*Wanting* to stop practicing FGM is not enough. The main empowering conditions allowing people *to enact their decision* not to have their daughters undergo FGM are: benefiting from social support (positive social capital), or being financially independent from the traditional solidarity network (sufficient economic capital). We therefore recommend finding ways to increase women’s/families’ empowerment to enact their decision not to practice FGM, mainly by: 1) providing them with new sources of social support, and 2) supporting them to gain more financial independence, including through schooling and improved access to better-paid employment.

**Conclusions:**

This study was the first to explore the experience of people who do not practice FGM in a context of high FGM prevalence and social pressure. The results and recommendations of this research can inform strategies for FGM abandonment and therefore contribute to improving or developing intervention strategies that promote the health and well-being of girls and women.

## Résumé

**Contexte:**

Les mutilations génitales féminines (MGF) peuvent nuire à la santé des filles/femmes. Plusieurs études ont identifié les déterminants socioculturels de cette tradition, or aucune n’a focalisé sur des personnes vivant dans un contexte où les MGF sont hautement pratiquées et qui refusent de faire exciser leurs filles. En Guinée, la prévalence des MGF est très élevée et la pression sociale pour faire exciser les filles est forte. Cette recherche a donc exploré les profils démographiques et socioculturels, et l’expérience de Guinéens qui ne pratiquent pas les MGF.

**Méthodes:**

Nous avons utilisé une méthodologie « d’ethnographie focalisée », et mené des entretiens individuels semi-structurés avec 30 femmes et hommes de différentes générations (jeunes adultes, parents, grands-parents) vivant à Conakry, en Guinée.

**Résultats:**

Nous avons constaté que les participants 1) ne divulguent pas tous leur statut de non-pratique, et 2) ont différentes expériences de pression sociale. Nous avons créé une typologie pour les décrire selon leurs différents profils et expériences, et les avons surnommés ainsi: 1) « activistes », 2) « discrets », 3) « courageux », 4) « stratèges ».

**Discussion:**

*Vouloir* abandonner la pratique des MGF ne suffit pas. Les principales conditions permettant aux participants de *mettre en oeuvre leur décision* de ne pas faire exciser leurs filles sont: bénéficier de soutien social (capital social positif), ou être financièrement indépendant du réseau de solidarité traditionnel (capital économique suffisant). Nous recommandons donc de trouver des moyens pour accroître l’autonomisation des femmes/familles pour qu’elles puissent mettre en œuvre leur décision de ne pas faire exciser leurs filles, principalement en les soutenant pour accéder à 1) du soutien social, et 2) l’indépendance financière, y compris par la scolarisation et l’accès à des emplois mieux rémunérés.

**Conclusions:**

Cette étude a été la première à explorer l’expérience de personnes qui ne pratiquent pas les MGF dans un contexte de forte prévalence des MGF et de pression sociale. Les résultats et recommandations de cette recherche peuvent éclairer les stratégies d’abandon des MGF et ainsi contribuer à améliorer ou à développer des stratégies d’intervention qui favorisent la santé et le bien-être des filles/femmes.

## Plain English summary

Female genital mutilation (FGM) can cause immediate and long-term health problems for girls and women. Many studies have identified the sociocultural determinants of this tradition, but so far, in a national context where FGM is highly practiced, none have focused on people refusing to have their daughters cut. As Guinea is a country showing one of the most prevalent FGM rates in the world, this research explored the demographic and sociocultural profiles of Guineans who do not practice FGM, as well as their non-practice experience in a context of high FGM prevalence and social pressure. We found that participants 1) do not disclose their non-practicing status in the same way, and 2) have different experiences with social pressure. We named the participants according to these experiences, as: 1) the “activists”, 2) the “discrete”, 3) the “courageous”, 4) the “strategists”. Moreover, our study reveals that *wanting* to stop practicing FGM is not enough. The main conditions allowing parents not to have their daughters undergo FGM are: benefiting from social support, or being financially independent from their traditional solidarity network. We recommend finding ways to support women/families by: 1) providing them with sources of social support that help them carry out their decision not to have their daughters cut, and 2) supporting them in gaining more financial independence, including through schooling and improved access to better-paid employment. These results and recommendations can contribute to improving or developing intervention strategies for FGM abandonment, and therefore promote the health and well-being of girls/women.

## Background

Female genital mutilation (FGM) is a modification of girls’/women’s genital area, performed according to sociocultural logic in the absence of medical justification [1]. The practice has the high potential of causing hemorrhage and infections, and interfering with normal physiological processes [1] which can lead to severe physical health complications (e.g., urinary problems, infections, difficult childbirth) and even death [2]. This painful and often traumatizing procedure [3] can also give rise to serious mental health predicaments (e.g., post-traumatic stress disorder, anxiety) [4]. It is estimated that at least 200 million women and girls currently alive have undergone FGM, and that more than 3.6 million additional cases of genital cutting occur worldwide every year [5, 6]. Beyond the sociocultural arguments put forward by practicing societies, FGM was never found to provide health benefits [1]. FGM is thus considered a violation of girls’/women’s rights to health, physical integrity, and life (when death results from the mutilation) by the international community [1, 7, 8]. Encouraging and supporting communities in the abandonment of the practice of FGM is therefore a global public health priority.

Despite a global reduction in the prevalence of FGM, strategies implemented to prevent the practice have had no visible effect in significantly reducing the prevalence in some countries [5]. One potential explanation is that some interventions might not be culturally appropriate [9]. Moreover, a large body of literature (e.g., qualitative research and ethnographic studies) has identified the cultural discourse justifying the practice of FGM in many countries [6], but has not focused on the sociocultural determinants of the *non-practice* of FGM, the actual desired output of public health interventions. In addition, many studies investigating the determinants of FGM have used descriptive/survey-type research designs with predetermined answer options, potentially leading to limited and superficial findings [2, 10]. There is thus a need to rethink research approaches and methodologies aimed at guiding the promotion of FGM abandonment.

### Theoretical background

This notion of “non-practice” of FGM is inspired by the concept of “positive deviance”, which involves people deviating from the prevailing sociocultural behavioral norm—i.e., for high prevalence countries, individuals deviating from the community expectation of perpetuating the FGM practice—and demonstrating positive health attributes as a result [11]. In this context, the term “deviance” is not pejorative [12]. Strategies using the “positive deviance” approach are based on the premise that solutions to problems arising in a given context already exist, and that community-inspired strategies are more likely to be culturally acceptable and feasible, and therefore sustainable [13, 14].

We therefore aimed to apply the concept of “positive deviance” for this study on FGM to gain an in-depth understanding of the sociocultural dynamics underlying this practice, to ultimately provide innovative avenues for informing public health actions promoting the abandonment of FGM in a culturally appropriate and sustainable way.

### Study population

To examine these questions, we looked at the case of Guinea, where the practice of FGM is widespread [15]. In fact, despite national efforts put forward for more than 30 years to discontinue the practice [9, 16], including the anti-FGM legislation [17, 18], FGM persists in Guinea with an almost universal prevalence of 95 to 97% [5, 15].

The most important reason for the practice of FGM in Guinea is respect for ancestral customs, followed by the objective of restraining women’s sexuality (before and during marriage), the purpose of which is to safeguard the family’s honor [9, 19]. The “positive attributes” of FGM therefore seem to be combined with its traditional nature, as the practice appears, for many people, to be deeply rooted and internalized [9], referring to Bourdieu’s notion of *habitus* [20]. Moreover, submission to the sociocultural norm of cutting girls is reinforced by social pressure, as well as by stigmatization and ostracism in cases of non-compliance [9]. In such context, it is virtually impossible to escape the practice without suffering negative social consequences.

Although in Guinea the practice may seem a priori unquestioned [21], Barry found in a recent anthropological study that nearly one third of Guineans have no intention of having their daughters undergo FGM, while others hesitate [9]. Yet, little is known about individuals and families who refuse to practice FGM in Guinea. Quantitatively, we do know that the prevalence of the practice of FGM slightly varies according to socio-demographic factors. For instance, Christians practice less compared to Muslims (77,9% vs 97,1%) [15]. Some variations also exist among ethnic groups, as those that practice the least are the Toma (69,3%), Guerzé (77,8%), and Kissi (88,2%) as opposed to the Soussou (97,9%), Peul (97,3%), and Malinké (95,9%) [15]. However, as per the latest data, FGM prevalence in urban areas (94,8%) is comparable to rural areas (94,3%), with the capital of Conakry showing a prevalence of 95,6% [15]. Qualitatively, Barry’s study states that families who do not practice FGM do not conceal their status, but do not advertise their decision either, and remain relatively invisible to the rest of the population [9]. However, knowledge about their beliefs, motivations, and justifications to go against the sociocultural norms, the context in which they live and their experience with social pressure is lacking.

Therefore, there is a need to understand the sociocultural determinants of the *non-practice* of FGM in the Guinean context of high prevalence and social pressure, in order to propose culturally appropriate strategies that would support families wishing to give up the practice. To address this knowledge gap, the research questions guiding this study were: 1) What is the demographic and sociocultural profile of members of families who do not practice FGM in Guinea?; 2) What is their lived experience with regards to not practicing FGM?; 3) How do they deal with the prevailing social pressure to preform FGM?; and 4) What conditions give them the ability to put into action their decision not to practice FGM, and thus act against the prevailing sociocultural norm?

## Methods

### Methodology

Health behaviors are often complex and deeply rooted in sociocultural contexts. In order to gain an in-depth understanding of the sociocultural logic underlying the non-practice of FGM in Guinea, we used the “focused ethnography” methodology [22]. In fact, given that the sociocultural dynamics related to the FGM practice are heterogeneous as per various factors (e.g., geographical regions) [6] and evolve over time [23, 24], we argue that it would require a colossal mobilization of resources (including time) to carry out *classical* ethnography prior to implementing each public health strategy in order for it to be adapted to its sociocultural context [9], since this type of exploration implies a long immersion in the field [25]. *Focused* ethnography is thus a more pragmatic approach as “this distinctive kind of sociological ethnography” [22] combines: 1) knowledge of the community under study by reviewing the literature, and by teaming up with local researchers (AD and HM in the present study) acting notably as cultural brokers; 2) shorter field visits along with the use of qualitative research tools (e.g., focused/semi-structured interviews); and 3) rigorous analysis of the collected material [26]. Besides, while these analyses included the use of numbers such as simple frequencies to detect patterns in the data [27, 28], this paper presents a subset of a broader focused ethnography approach.

### Conceptual framework

We used three types of concepts to explore what conditions and forms of power are required for enacting the decision to not practice FGM: 1) total capital; 2) interdependence vs individualism; and 3) resilience. The concept of “capital” refers to the objective or subjective benefits that individuals can have access to within their social environment, allowing them to gain power; **total capital** is the accumulation of various sources of power that may vary according to context [29]. This notion includes the cultural, social, and economic forms of capital. **Cultural capital** applies to the acquisition of assets related to culture; this can refer to education, knowledge and intellectual skills, or take the form of culturally valued objects. More specifically, we considered *institutionalized* cultural capital, which consists in the acquisition of power through academic qualifications [30]. For this study, we therefore used participants’ educational level to determine their degree of access to institutionalized cultural capital (which we thereafter refer to as “cultural capital” for the sake of brevity). **Social capital** pertains to having access to social networks within which support is provided on a daily basis and during challenging times; this solidarity system also includes respecting and supporting one’s decisions. For this study, we explored whether participants had social support for not practicing FGM (referred to as “positive social capital”), or if they experienced social problems because they decided not to practice FGM (referred to as “no social capital”). **Economic capital** refers to financial power, and thus the corresponding independence that allows individuals to make their own life decisions. For this study, we used households’ basic goods possession or services^1^ and housing occupancy rate^2^ as proxy to assess the economic level of participants’ family.^3^**Interdependence** is defined as a “traditional” family or clan solidarity system, which involves a form of social protection and, in some cases, access to funding sources; this connectedness to others often implies lineage expectations [31]. In opposition, **individualism** can be defined as the emancipation of the person from the values of her/his family or community, and as the independence and self-reliance of the person, who can evolve in separate and heterogeneous social fields [31]. Finally, the psychological component of **resilience** consists of the ability to regulate emotions while coping with adversity, to adapt, and to achieve well-being [32].

### Sampling and recruitment of participants

Recruitment was focused on Guinea’s capital of Conakry, as we anticipated more potential “positive deviants” could be reached in a limited amount of time in such an urban milieu, thus maximizing the feasibility of our study. We recruited participants through “snowball sampling” [27], asking local organizations promoting FGM abandonment, community members (via informal conversations), and eventually study participants, to point us to potential participants. This method proved to be effective as “positive deviants” belong to a rare and concealed sub-population that may be hard to identify [33]. Attention was paid to ensure that the sample was of “maximum variation” [27], that is, participants were selected as much as possible according to a heterogeneity of factors, namely, generation (i.e., young single adults (18–30 years old); parents (including mothers-in-law/co-spouses or aunts); and grandparents), as well as socio-demographic variables (i.e., education level, ethnicity, etc.). Since in Guinea FGM is usually practiced between birth and the age of 15 [15], participants could be included in our study if they belonged to families with at least one uncut girl aged 16 or over, or girls under 15 years old with the firm parental assertion that they will not be cut. Young adults who were not yet parents could participate if they expressed a clear conviction against the practice. Our sample could include participants who were themselves cut. Minors (aged less than 18 years old [34]) were not offered to participate, but no other exclusion criteria were applied in the recruitment of participants/families. Given our limited budget, enrolment of participants ended after the inclusion of 30 people.

### Data collection

As one-on-one discussions are generally more appropriate for exploring sensitive topics such as FGM [25], semi-structured, in-depth individual interviews were conducted. The interview schedule included: 1) a narrative (unstructured) part, to let participants express themselves freely on their personal experience relating to the non-practice of FGM in Conakry’s sociocultural context; 2) the impacts of belonging to a non-practicing family on participants’ social capital; 3) the resilience story of participants dealing with problems because of their non-practicing status; and 4) socio-demographic data, pertaining to: age; education level; religion; ethnic identity; occupation; and family’s economic level.

Interviews took place in private spaces at the convenience of participants, so they could feel free to express themselves without being heard by their family or entourage. Discussions were carried out in French or in the language participants were most comfortable with, and lasted an average of 47 min. All interviews were digitally recorded. Data collection was carried out during the months of January and February 2019. French verbatim were literally transcribed by an independent professional transcriber, and interviews that were conducted in local languages (Soussou, Malinké, Pular) were transcribed into French by HM. Any data transferred through the Web was sent encrypted to insure the security of collected material [35].

### Data analysis

We used the “Framework analysis” method to analyze data, as this approach: facilitates the overview of each case; enables comparisons and contrasts between participants’ experiences, points of view and living situations; and helps identify key themes, as well as reveal patterns and typologies in the data [27, 36]. This five-step method consisted of 1) “familiarizing with the data”, by listening to audio files and reading transcripts; 2) “identifying a thematic framework”, drawing on the themes emerging from the data; 3) “indexing”: coding all verbatim in a systematic way using the MAXQDA software; 4) “charting”: 4.1) producing case-by-case summaries according to the main themes, and 4.2) displaying all cases in a matrix; 5) “mapping and interpretation”: 5.1) intra-case analysis, and 5.2) inter-case analysis, comparing and contrasting all cases for each theme to unveil typologies and patterns in the data. This last stage also implied critical analysis to derive recommendations for informing policies and strategies. This interpretation of the results was based on theoretical knowledge as well as on researchers’ experiences and observations of the sociocultural context.

### Reflexivity and positionality

While informing potential participants about the study objectives, the interviewers (MHD and HM) specified that the ultimate goal of the study was to make recommendations, based on their narrative, for strategies promoting FGM abandonment in Guinea. However, we do not believe that the participants’ responses were notably biased (social desirability) because of this statement, given that many of them were already active in the fight against FGM in their daily reality, and that the responses of the remainders were consistent with those of the activists.

Moreover, MHD being a white Western woman, positioned herself as an “outsider”. This legitimized asking participants to explicit their experiences and views, as they could have been tempted to imply cultural meanings if they would have solely been talking to a Guinean interviewer, because of taboos surrounding FGM or taken-for-granted local knowledge [25]. Complementarily, HM being a Guinean was an “insider” who facilitated reaching out to potential participants and establishing a trusting relationship [25]; she also conducted interviews held in local languages. This “mix” of positionalities was therefore an asset that enhanced the depth of the data collected.

### Trustworthiness

Different measures were used to insure credibility of findings. The narrative part of the interviews started with the question: “When did you hear about FGM for the first time?”, allowing participants to immerse themselves in the story of their own experience in a more natural and chronological way, and to share the evolution of their reflections over time. Verbatim were verified for transcription accuracy by MHD; translated interviews were verified for accuracy of translation and sociocultural significance [25] by an independent research assistant, and thereafter by MHD. During the “charting” phase, MHD entered the coded data in the matrix, and performed quality checks with raw data [27]; HM confirmed the procedural adequacy of the data displayed in the matrix [27]. The “simple count” technique allowed to confirm patterns found in data, as opposed to anecdotal accounts [37]. Interpretation of findings was first performed by MHD, and thereafter validated in several ways, through: verification by the Guinean co-researchers acting as “cultural brokers” (AD, HM); “expert checking” [38]; verifying in the literature for congruence with similar phenomenon [27]; analyzing plausibility of results [27]; and inclusion of raw data in our scientific paper to support findings and interpretations [25].

### Transferability

Given that the results were validated for cultural adequacy, we are of the opinion that our recommendations are transferable to Guineans who wish to abandon the FGM practice and who are living in Conakry, as well as in other Guinean urban settings [25, 27]. Furthermore, we believe our findings and recommendations could be applicable to other Guineans and West-Africans wishing to abandon FGM, but we recommend testing and verifying this assumption [25, 27].

### Reporting

We used the SRQR reporting guidelines [39].

### Ethical considerations

This study received ethical approval from the Ethics Review Board of McGill University as well as from the National Ethics Committee for Health Research of the Republic of Guinea. Researchers obtained the free consent from participants after explaining and having them read the detailed consent form. Moreover, participants were all asked for their assent before recording the interviews. No minor was interviewed. All interviews were anonymized (a code was assigned to interviews). Finally, data is stored in MHD’s computer, which is protected by a password.

## Results

Eighteen women and twelve men agreed to take part in our study, for a total of 30 people aged 18 to 69 years old and representing three generations: young adults (18–30 years old), parents, and grandparents (Table 1); no “young adults” had children, except for one single woman. Participants were generally enthusiastic about participating, and some even volunteered to be interviewed without being solicited, having heard about our study by members of their network. Three persons refused to participate, fearing: the interview would be broadcast on the radio; to be accused of “following the Whites” by the surrounding population; and as an imam, he would be judged by his followers. These refusals occurred despite explaining that the interviews would take place in a private place of their choice and would be anonymous, and that audio recordings would only be used for the purposes of the study.

**Table 1:** Participants’ demographic characteristics (*n* = 30)

		*n*	Percentage (%)
Generation	Grandparents	4	13
	Parents	18	60
	Young adults	8	27
Sex	Women	18	60
	Men	12	40
Level of education	University – doctorate	1	3
	University – master	8	27
	University – bachelor	5	17
	*Currently students (secondary school)*	*5*	*17*
	Secondary or professional training	6	20
	Primary or no education	5	17
Religion	Muslim	24	80
	Christian	6	20
Ethnic identity	Malinké	9	30
	Peul	6	20
	Badiaranké	5	17
	Soussou	4	13
	Kissi	4	13
	Guerzé	1	3
	Manon	1	3

### Demographic and sociocultural profiles

Our sample consisted mainly of highly educated people, with almost half of them (14/30) having a university degree (Table 1). Most participants were Muslims (24/30). Interviewees represented a relatively wide range of ethnic groups, but nearly one third of them were Malinké (9/30). While all participants shared the conviction that FGM is harmful, only one of them—a young man native to a Senegalese region where FGM is not practiced—stated coming from a family that has not practiced FGM for many generations. This confirmed that essentially all participants came from a culture where FGM was the norm. Only 3/18 women had not undergone FGM themselves. Aside from the above-mentioned young man, all male interviewees (11/12) mentioned being married to a cut woman, or having cut sisters.

### Experience with not practicing FGM and dealing with social pressure

**Fig. 1:**
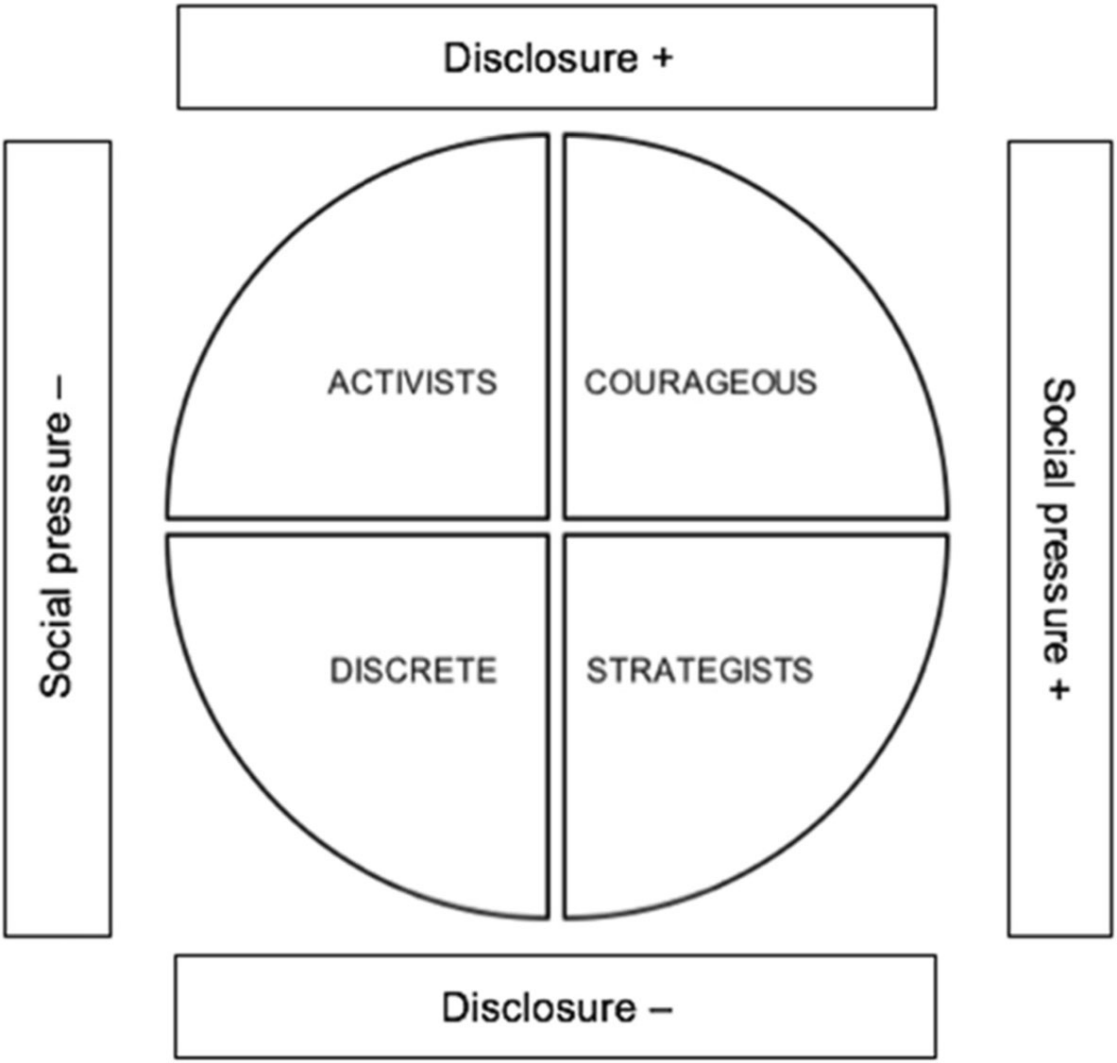
Level of disclosure of not practicing FGM vs experience of social pressure. The “activists” openly speak about being against FGM, and do not fear/perceive social pressure. The “discrete” avoid social turmoil by not disclosing their decision, but do not hide it at all costs either. The “courageous” openly speak about being against FGM, and suffer corresponding social consequences. The “strategists” avoid social turmoil by deceiving/lying about their decision

Firstly, participants revealed distinctive patterns regarding their attitudes and behaviors in terms of the degree to which they disclose (or not) their non-practicing status in their daily lives. Some people said they fully assume their decision not to have their daughters cut, and even promote the abandonment of girls’ genital cutting. Others mentioned they do not advertise their non-practicing status, but do not hide it at all costs either. And some confided they not only hide their status, but use “deception”, in other words, they lie to those they consider threatening by pretending to have their daughters excised. Secondly, it was found that participants’ perception of being subjected (or not) to social pressure to have their girls cut was varied, and even polarized. Indeed, some said they were not under pressure or stated they were not affected by societal expectations. Others disclosed undergoing heavy social consequences for not conforming to the norm. The remaining explained how they managed to avoid social turmoil: either by being discrete, or by using “deception”. A typology was thus created to describe participants as per their various profiles and experiences, which we named as: 1) the “activists”, 2) the “discrete”, 3) the “courageous”, 4) the “strategists” (see Fig. 1). The commonalities and singularities pertaining to their cultural, social and economic capitals are shown in Table 2,^4^ followed by the description of their experiences.

**Table 2:** Summary of participants’ characteristics according to typology (*n* = 30)

		Activists	Discrete	Courageous	Strategists	Total
n		14	12	2	2	30
Social capital^a^	Yes	14	12	0		26
	No	0	0	2	2 (partial)^b^	4
Cultural capital	University – doctorate	1	0	0	0	1
	University – master	4	4	0	0	8
	University – bachelor	2	3	0	0	5
	*Current students*	4	1	0	0	5
	Secondary or professional training^c^	1	2	1	2	6
	Primary or no education	2	2	1	0	5
Economic capital	High	10	9	1	1	21
	Middle	4	3	1	1	9
	Low	0	0	0	0	0
Type of social ties	Individualistic discourse	13	7	0	0	20
	Interdependence discourse	0	0	2	1	3
	*Missing data*^d^	*1*	*5*		*1*	*7*

^a^Social support for not practicing FGM

^b^The “strategists” perceive that some of their entourage do not support the non-practice of FGM

^c^Professional training, such as midwifery or technician certifications

^d^The narrative did not allow to classify in one or the other

#### The “activists”

The fourteen participants classified as “activists” stated they speak openly about their position of being against FGM, and for those who are parents, of not having their own daughters cut. Advocating against FGM is either part of their work or something they do with others around them.

*“I decided to fight [the practice of FGM]. The question for me is not anymore if I will practice it, no, that is bygone; but it is to fight the practice*” (father, master level, works as an activist in an NGO).

They also all confirmed not experiencing problems relating to social pressure, and living in a favorable social context allowing them to enact their decision not to have their daughters cut. Moreover, they were not concerned about being criticized.

*“Before, [what people said about me not being cut] bothered me, because when you're a child and you [are] in an environment where you are with friends and it is you who is [criticized], it is disturbing. But today [...] I am proud [of not being cut]”* (young uncut woman, bachelor level).

This subsample included many people having high levels of education and economic capital (Table 2). Their occupations ranged from students to NGO managers, legal experts (lawyer and jurist), healthcare provider (midwife), teacher, and commerce workers (cashier and salesperson). Additionally, all of them expressed individualistic values in their narrative, such as:

*“They are my children, I do not have to receive orders from anyone”* (father, master level).

*“I am never going to listen to what people say to the point of sacrificing my child. If you want, continue to mock me. But I will not listen to you for you to kill my child”* (mother, no education).

Some parents said they must protect their daughters from certain family members—commonly paternal aunts and grandmothers—who could potentially “steal” their children to have them cut behind their backs.^5^

#### The “discrete”

Twelve participants stated they do not openly talk about their decision not to have their daughters cut, except to a group of people (immediate family or friends) with whom they share common values.

*“This decision, I did not make it public. We are in Africa, everyone has his way of thinking. Me, my decision is that I do not want to excise my girls. That’s it*” (father, master level).

However, they did not actively try to hide their status at all costs. They also all stated having no social problems regarding their decision, or said they were not susceptible to social influences.

*“Some may say what they think about it, but it does not bother me [...] because I can convince them why I decided, but they cannot convince me why they do it [...]. We are peaceful [with our decision]”* (grandfather & father of uncut girls, master level).

Very few interviewees explicitly stated that their discretion of not publicizing their point of view was to avoid social problems.

*“I [used to] advise women [...] to stop [practicing FGM]. But every time you approach this subject matter, if there are old women, they will look at you as if you are an ignorant, damn, against our customs, uprooted, lost,... You see... So finally, [I] do not talk about it anymore”* (aunt, master level).

Some “discrete” also alluded to the theft of girls’ phenomenon. Some said they themselves were “stolen” from their mothers to be cut when they were children. Others warned their extended family not to take their daughters to have them excised, otherwise they would report them to judicial authorities.

Other participants expressed that nowadays in Conakry, a growing proportion of people are not interested in the topic of FGM and do not discuss it anymore, as if for some it was a solved issue from a past era, or if it had become more of a private matter.

*“Nobody talks about this practice. [...] People do not ask me [if I was excised]. I have never been asked that”* (young uncut woman, bachelor level).

An important proportion of this subgroup included people with high or very high levels of education, and mainly high economic capital (Table 2). They were either engineers (4/12), healthcare providers, working in the commerce industry (e.g., cashier, hairdresser), housekeepers or students. One participant was looking for employment. Two elders were retired. Also, most of their narratives reflected individualistic values.

*“It is you who must make a decision to say that your daughter will not be cut because she is your daughter, no one else should force you. Me anyway, I decided that my daughters will not be excised, it’s my decision”* (mother, primary school level)*.*

#### The "courageous"

Two interviewees—a mother and a grandmother—revealed that as soon as the people around them knew their daughters would not undergo excision, they were criticized and rejected. This caused them to suffer greatly, as they were living in a social environment that was highly hostile to those who dare not conform to the FGM tradition.

*"One day, my in-laws told me to send my first daughter to have her excised, I refused. They insulted me; [When] my daughters approach [my neighbors] they say: "Stay away from us, those who are not cut have to go to the other side. If a person is not excised, it is an animal"* (mother, primary school level).

*“Eeeeh!! They were mad at me!! They called me “crazy”. [They said:] “She does not need to live, she’s going to see.” [...] I was not going to anybody’s home, nobody came to my house [...] I felt... frustrated, I felt isolated”* (grandmother, professional school).

The mother particularly insisted on constantly having to keep an eye on her daughters so that they are not “stolen” by relatives to be excised. They expressed trying to be brave despite adversity, relying on their faith, and above all, feeling peace of mind for doing what they consider to be right for the girls, thus showing a high degree of resilience.

*“I even divorced with my husband, so much that I had social pressure. But! I stayed strong. I was told that I am a hard woman. Ah! ah! What I do is for the future of children”* (grandmother, professional school).

Moreover, not only do they openly assume their decision not to have their daughters cut in spite of the heavy social cost they must endure, they even testified advocating for the non-practice of FGM: “*If I see someone who does it, I will advise her to stop by telling her the consequences”* (mother, primary school level). The eldest even founded an association to save girls from excision at a time when social pressure was even more intense than today. We therefore classified them as being “courageous”.

Furthermore, the mother spontaneously confided that in addition to being sidelined by her lineage, she has been abandoned by her husband for 5 years because of her decision to avoid FGM for their daughters; she thus had to take charge of all the expenses for her daughters and herself. Despite her moderate economic capital and financial worries, her income as a shopkeeper (restaurant) was sufficient enough to allow her to be fully economically independent. The older lady was a retired midwife with a high economic capital. None of these interviewees expressed individualistic values.

#### The “strategists”

Two mothers adopted the strategy of concealing the truth about their decision to not have their daughters cut, because they believed they would otherwise face significant problems arising from their social environment.

*“When they say to have your daughter excised, you answer that [...] her paternal aunt will take care of it. [...] You send her to the healthcare center, and then you come back to tell the neighborhood she has been excised: who will tell you to spread her legs to check? Nobody”* (mother, secondary school level).

*“They will force me to do it. So I'm going to [...] deceive the appearance, but I would not do the practice. [...] I will not say it, even to my husband I will not say it. [...] [It’s] the easiest way to avoid problems, but to stop anyway, to giving up excision”* (mother, professional school).

The motivations underlying their use of “trickery”^6^ are manifold. First, they explained that beyond sparing their daughters from being cut, they make sure to prevent them from being “stolen” by a relative to undergo cutting unbeknownst to them. Second, they protect their uncut daughters from being stigmatized and ostracized by their peers—thus protecting the girls’ social status. Third, mothers act this way to spare themselves from the social pressure to comply with the tradition. And last, while they both said they benefited from support for their decision within a very restricted social circle, they had to use deception with specific people to protect their own social capital (e.g., to protect their marriage).

These women had moderate educational levels and lived in high socioeconomic contexts (Table 2); but while the woman hiding the truth from her husband is working in the commerce industry, her narrative does not allow us to find out if she could be financially independent in case of divorce. The other woman was a housekeeper. None of these participants expressed individualistic values, suggesting they live in a context of interdependence.

This stratagem is acted out in various ways, as seen in the following examples. Some take their daughters to the healthcare center to ask the provider to pretend to perform excision, or to do a light incision on the girl’s genital area so she would believe she was cut: this way, mothers insure their daughters would not reveal they are uncut, preventing them from being “stolen” to undergo the procedure. Others have their daughters wear the traditional post-excision garments (a colorful loincloth knotted over their chest), to “deceive the appearance”.

While very few interviewees stated they use “trickery”, many participants and people met informally alluded to this strategy. Among them, a grandmother we classified as “discrete”—since she is currently discreet about her granddaughters’ non-excision status—explained she used the deception strategy to spare her younger daughter from excision. It seems to be transiently used, until the apparent social norm shows a trend towards the non-practice of FGM.

## Discussion

Our data confirm an apparent culture supportive of the FGM practice in Guinea and the persistence of social pressure in favor of FGM continuation—as seen with the experience of the “courageous” as well as with the need of the “strategists” to use deception to avoid suffering from such pressure—occurring most probably within the least educated and privileged strata of the population. It also confirms the existence of a punishment system, in the form of stigmatization and exclusion, aimed at promoting compliance with the FGM tradition. Some people might indeed not feel able to cope with the high social cost of being stigmatized and ostracized in case of hostile social environment, and therefore yield and have their daughters excised. These findings correspond to the description of the operating modes of traditional collectivist societies, which are characterized by the subordination of individuals’ personal interests in favor of the welfare of families/collectives [40, 41]. In such a context, people belonging to social categories that are considered to be inferior—usually women and children—often suffer from various forms of “ordinary violence” [42], like FGM. Our findings are also in line with social norm theory and particularly with social convention theory, which posits that individual actions are conditioned by interdependent expectations [43–45].

Yet, echoing Barry’s study [9], we too easily found people not practicing FGM (who were even enthusiastically willing to participate in our study), and we argue that this could indicate that change is beginning to happen in Guinean society towards the abandonment of FGM, at least in Conakry’s urban context. Indeed, as rural societies are typically characterized by a limited number of social group options, it is usually easier to “control” community members and preserve traditions [40]. In contrast, urban environments are characterized by a greater diversity of cultures, thus facilitating individualization processes and allowing people to distance themselves from traditional practices perpetuated through social relations [31, 40]. This urban-rural divide is documented for the practice of FGM, which is generally less prevalent in cities than in rural areas [6]. Even if this trend is not statistically present in Guinea [15], it is likely that living in the Guinean capital may offer opportunities for its inhabitants to abandon the FGM practice. But apart from the mere fact of living in an urban context, favorable conditions—such as schooling and financial autonomy—are generally required to reach emancipation from the shackles of sociocultural dependency [31] when collective change does not seem to be foreseen and therefore has to be made on an individual basis.

Furthermore, an unexpected and important finding in our study is that most interviewees (the “activists” and the “discrete”) stated they experience no significant problem relating to the pro-FGM social pressure. Barry’s very recent work (2019) indeed shows that nowadays, almost half of women living in Conakry (46%) perceive that no social sanction results from non-compliance with the FGM tradition, while a lesser proportion (29%) considers that social pressure on families still occurs [19], suggesting a decline in social pressure. However, the conditions allowing this shift were not investigated. This may be due to the above-mentioned hypothesis that attitudes are gradually changing in Guinea with regards to FGM. But in spite of this emerging trend, and despite the fact that many Guineans would prefer not to have their daughters cut [9], the FGM prevalence still remains high even in the youngest generations of Guinean girls, as 92% of the 15–19 year-olds are cut [15]. However, uncovering the “deception” trend leads us to question Guinea’s high FGM prevalence, as our data indicate that FGM might be over reported to conform to the apparent social norm. This “trickery” phenomenon was also observed in Mali: physical examination by qualified healthcare professionals revealed that the genitals of many girls who were declared excised were in fact intact; mothers of these girls finally admitted having made this “false statement” to prevent their daughters from being targets of mockery from their peers, given the conflict of social values.^7^

Moreover, the fact that we did not find more people with the “courageous” profile may be because few Guineans benefit from the empowering conditions that enable them to turn their intention not to practice FGM into reality. Following is a discussion about the profiles and specific sources of power accessible to our participants according to their typology, which enable them to act against the prevailing FGM norm.

Besides their willingness to publicly divulge their non-practicing status or not, the profiles of the **“activists”** and the **“discrete”** are very similar, as they do not experience problems with social pressure, and show an accumulation of different forms of capital enhancing their total access to power. They generally display a high cultural capital through their high level of education. This in turn increases their chances to access to better employment (e.g., engineering) and corresponding high salaries, as evidenced by the high economic capital shown by most of them. We posit that it gives them the assurance of having the capacity to be financially independent from their lineage if needed, thus feeling free to act as they intend to. Moreover, most of them have also made statements demonstrating a propensity towards their individualization, showing a determination for being independent from their social networks and lineage in case of disagreement around the issue of the non-practice of FGM. But above this, they all mentioned being part of social network(s) that allow them to enact their decision not to have their daughters cut. They live in social niche(s) in which people share comparable values and attitudes regarding FGM, thus sparing them from being ostracized or stigmatized.

The **“courageous”** present a lower total capital, i.e., a low/medium cultural capital and most importantly a blatant lack of social capital, given the very strong social pressure to perpetuate the FGM practice that prevails in Guinea. Nevertheless, they have the capacity to carry out their decision not to have their daughters cut through their economic capital, as their relative economic independence empowers them to take the high financial risk of acting despite living in a context of interdependence and being abandoned by their lineage. This suggests that benefiting from a minimum threshold of economic capital could be one of the pivotal conditions allowing Guineans living in Conakry to enact their decision, as it provides them with the individual means for insuring their subsistence and independence. Their high degree of resilience also allows them to assert their position despite the weighty social cost they must endure.

The **“strategists”** use what some might call a “ruse” [31] to avoid two types of pitfalls: 1) to remain in solidary with their lineage and thus submit their daughters to excision (which is not an option for them), and 2) to declare their non-practice and therefore be marginalized, ostracized and stigmatized (which would amount to a “social death”). Their dissimulative strategy is based on the assurance that no one will physically verify if their daughters were actually cut. Like the “courageous”, they exhibit a lower total capital, having an intermediate cultural capital as well as limited social capital—with some people closely tied to them perceived as threats to their daughters’ physical integrity. They therefore “do the math” that the social—and most probably financial—cost of asserting their decision would be too high, with consequences (such as divorce) being beyond what they could bear. Despite their middle to high economic capital, it is not clear, based on their narratives, if these mothers could have financial independence from their husbands and families.

As a side note, we would like to point out that according to their testimonies, a slight incision meant to deceive is not considered by our participants to be a form of FGM. They indeed stated that they do not practice FGM and found ways to protect their daughters from this tradition. But such an incision—however light—by definition falls under the category of FGM type IV [1]. Moreover, the fact that healthcare providers perform FGM is considered as medicalization of FGM, a phenomenon that is on the rise in Guinea [6, 9] although illegal [16, 17]. By extension, it could be interpreted by some that the medicalization of FGM is one of the means that “strategists” use to avoid social pressure, but based on our findings, we do not see it that way. We however argue that this “slight incision” method used to protect girls from a more invasive form of FGM should not be encouraged.

In sum, not wanting their daughters to be cut is not enough. In order to enact this decision in a sociocultural context of quasi-universal FGM practice, parents must rely on significant empowerment conditions to survive and thrive, and we posit that the following are the main sources of power.

**1. Favorable social capital**. We are of the opinion that since enacting the decision to not have girls undergo FGM is a social issue in the first place, the solution is therefore primarily social. Indeed, parents that decide to abandon the tradition need to benefit from a strong social capital that supports the non-practice of FGM, which includes sharing similar viewpoints on and criticisms towards the tradition of FGM with significant people in their families/network [46]. Moreover, this network inevitably has to involve both the mother and the father, and should preferably also include the extended family—especially paternal grandmother and aunts. Or alternatively, families wanting to abandon FGM need to live in social environments where people are not interested in knowing whether excision is practiced or not, as seems to be increasingly the case in Conakry.

**2. Sufficient economic capital allowing financial independence**. In the absence of favorable social capital, parents need to have sufficient economic capital so they can act individually and be financially independent if their lineage cuts off its financial support. Our sample of “positive deviants” included people with middle to high economic capital, and no families with a low socioeconomic status. As Guinea is known to be one of the most disadvantaged countries in the world [47], individuals in a survival state might have very limited options and therefore be in a situation where they are unable to act independently of their traditional solidarity networks [31]. Economic capital could also be a lever to enact the decision in cases where parents of uncut girls who financially support other family members who are pro-FGM, can threaten to cut off this support and therefore force the latter to respect their parental decision [48].

While there is a scarcity of studies assessing the economic repercussions of the FGM practice on families and societies [49], there is a blatant research gap regarding the economic implications for families who chose not to practice FGM in high prevalence countries. However, it is known that when more vulnerable individuals increase their financial empowerment, they can free themselves from normative expectations, as economic independence contributes to restructuring domination relations [42]. Working at reducing inequalities in accessing social, cultural and economic capital is therefore essential to achieving FGM abandonment in Guinea, in particular for women. This recommendation is consistent with the “modernization theory”, which postulates that increasing social factors at the population level such as schooling and employment will contribute to FGM abandonment, most probably through an individualization process [2, 50].

**3. Other forms of resilience**. Parents who do not benefit from a supportive social environment need to rely on other sources of strength to cope with social pressure, which often comes in the form of stigmatization and ostracism. This “positive response to adversity” can take various forms [32]. Some people might find some serenity in the thought of protecting girls’ well-being, based on the knowledge that FGM is a harmful practice. Others might rely on their faith, spirituality or religious practice to endure these challenges. Some might draw support from individual people, social networks, or community resources.

**4. Protecting daughters from “theft”**. Many parents need to constantly be vigilant to protect their daughters from the risk of being “stolen” to undergo cutting—a phenomenon that is unreported in scientific literature [51] but well-known by Guineans. In a collectivist society like Guinea, children do not belong to parents, but are part of a lineage in which paternal aunts and grandmothers have the tacit responsibility to ensure norms and values are respected [52]. This logic does not take into account parental decision, and parents have to find ways to safeguard their daughters. While some mothers use “deception” to avoid this kind of situation, we argue that this is not a viable solution as it is based on a lie rather than on a profound and sustainable change in the societal mentality with respect to maintaining girls’ physical integrity.

### Recommendations for the abandonment of FGM in Guinea

Based on our findings, we suggest the following strategies to support parents to put into action, in a sustainable way, their decision not to have their daughters undergo FGM.

**1. Increasing individuals’/families’ social capital.** Strengthening existing associations or developing new community resources dedicated to providing people/families who do not practice FGM with sources of social capital should be a priority, in order to support those who cannot count on family or community networks to help them safely and freely enact their decision. This “restructuring” of solidarity systems in Guinea [31] would allow non-practicing people/families to bond with others who share common views and values, provide them with new sources of social support [9], and thus break their isolation and singularity. And importantly, these social capital opportunities would allow additional people/families to increase their agency and empowerment [53, 54], allowing them to enact their intention of stopping the practice of FGM.

**2. Favoring mothers’/families’ economic capital.** Supporting people in gaining adequate economic power is also an important strategy that increases people’s—especially women’s—ability to carry out important decisions [54, 55] such as abandoning FGM. Guineans having to circumvent the traditional modes of solidarity should have means to gain financial independence if necessary. This could be done through 1) financial support provided by associative networks [31]; 2) improving people’s access to employment; 3) for the long term, ensuring the population attains higher education levels, which in turn contributes to access to better-paying employment. This is even more urgent for marginalized and isolated women who are solely responsible for their own needs and those of their children, especially since Guinean society is characterized by discrimination and injustice towards women stemming from sociocultural prejudices [56]. We therefore argue that NGOs and associations working at promoting FGM abandonment, as well as those working at increasing Guinean’s social and economic capital, work together in a concerted way along with relevant governmental ministries.

### Limitations of the study

Although we aimed at including people with varied sociodemographic profiles, our sample ended up being characterized by numerous highly educated and financially privileged participants, with no participants from a low level of economic capital. We might have missed some less educated and underprivileged people because of our snowball technique and the time constraints imposed by our study (doctoral research with limited funding). Therefore, we could not capture the perspective of some “positive deviants” who might have existed in Conakry. Moreover, the “courageous” and the “strategist” subsamples represented only a small percentage of study participants, but nevertheless clearly displayed distinctive profiles and experiences. Another limitation of our study is that we did not include the symbolic capital in our conceptual framework and our interview guide—which refers to participants’ prestige, reputation or pride provided by particular accomplishments or status (e.g., highly valued profession) [57]. Therefore, we were unable to determine whether this form of capital had an impact on participants’ ability of to enact their decisions.

Despite these limitations, one strength of our study is that the focused ethnography methodology was an efficient, effective and innovative approach that allowed us to better understand under which conditions Guineans living in Conakry enact their decision to not practice FGM. Also, this study is unique in providing a rich understanding of the experiences and sociodemographic profiles of “positive deviants” who have not been given full attention in previous studies. Finally, by putting forward the conditions needed by Guineans to enact their intention not to have their daughters cut, our study allows the production of locally relevant recommendations for public health strategies.

### Perspectives for future research

It is crucial and urgent to replicate this study in rural and forest areas of Guinea, as the lived experiences of people who do not practice FGM might differ from what was seen with our participants living in an urban context. Attention should also be paid to include more underprivileged and less educated people, to better explore what would be the optimal conditions allowing them to abandon FGM. Moreover, although the “deception” strategy also seems to be very well known in Conakry, it is virtually undocumented in the scientific literature,^8^ and scarcely discussed in the grey literature [48]: its magnitude is consequently unknown. This phenomenon should be further explored in future studies. Finally, as our study did not allow us to discover if participants’ symbolic capital was involved in their capacity to enact their decision, it would be worth further exploration.

## Conclusions

Our results suggest that *wanting* to stop practicing FGM is not enough. The main condition for parents *to enact their decision* in Conakry’s sociocultural context is to be able to rely on social support network(s) that are favorable to the non-practice of FGM. Otherwise, mothers and families must have the capacity to act individually from their traditional solidarity network, which essentially revolves around being financially independent (sufficient economic capital). In sum, Guinean mothers/families living in Conakry need to gain more social and economic capital, and intermediately more cultural capital, to empower them to have control over their daughters’ bodily integrity.

This was the first study to explore the experience of family members who do not practice FGM in the context of Guinea’s high prevalence and social pressure. The results and recommendations of this research will inform strategies for FGM abandonment and therefore contribute to better intervention that protects the health and well-being of girls and women.

## Data Availability

The datasets generated and analyzed during the current study are not publicly available, to maintain the anonymity of the persons interviewed. However, sufficient anonymous verbatim extracts are presented in the paper to illustrate the results.

## Abbreviation

FGM: Female genital mutilation

## Footnotes

1 The sum of the following assets: radio; television or computer; telephone (landline or mobile); refrigerator; stove (electric or gas); table, chairs or bench; armoire or library; mattresses; electricity; running water; modern toilets; and finished floors (tiles or cement). An arbitrary degree was thereafter assigned: a total of 10–12/12 was considered as “high”, 6–9/12 as “middle”, and 0–5/12 as “low”.

2 The number of occupants divided by the number of rooms. An arbitrary degree was subsequently assigned: ratio of 0–1 person/room = “high”, 1–2 persons/room = “middle”, more than 2 persons/room = “low”.

3 The overall economic level attributed to the family was an “average” of these two indicators (e.g., high + high = high; middle + low = middle).

4 No patterns were found in narratives about a possible relation between interviewees’ religious or ethnic identities and enacting the decision to not practice FGM.

5 They used the French verb “voler” (“to steal”), which could be defined in this context as a temporary “theft” for the sole purpose of subjecting the girl to excision to meet sociocultural norms.

6 They used the French word “tromperie”.

7 Pr. Touré M: Personal communication (2019). This finding is not officially published, as it was fortuitously discovered.

8 Barry referred to families who “pretend” excising their daughters, but did not describe this phenomenon further [9].

## References

[1] World Health Organization. Female genital mutilation (fact sheet). 2020. https://www.who.int/news-room/fact-sheets/detail/female-genital-mutilation. Accessed 4 Feb 2020.

[2] Andro A, Lesclingand M (2016). Female genital mutilation. Overview and current knowledge. Institut National d’Études Démographiques. Population.

[3] Institut National de la Statistique (INS) et ICF (2018). Enquête démographique et de santé en Guinée 2018 : Indicateurs clés Conakry, Guinée, et Rockville, Maryland, États-Unis d’Amérique.

[4] Mulongo P, Hollins Martin C, McAndrew S (2014). The psychological impact of female genital mutilation/cutting (FGM/C) on girls/women’s mental health: a narrative literature review. J Reprod Infant Psychol.

[5] UNICEF (2016). Female genital mutilation/cutting: A global concern.

[6] UNICEF. Female Genital Mutilation/Cutting: A statistical overview and exploration of the dynamics of change. New York: UNICEF; 2013. 186 pages.

[7] African Commission on Human and Peoples' Rights. Protocol to the African charter on human and peoples' rights on the rights of women in Africa; 2003. 32 pages.

[8] Organisation of African Unity. African charter on the rights and welfare of the child. Addis-Abeba; 1990.

[9] Barry AAB (2015). La perpétuation des MGF en Guinée – Analyse socio-anthropologique des déterminants.

[10] Doucet MH, Pallitto C, Groleau D (2017). Understanding the motivations of health-care providers in performing female genital mutilation: an integrative review of the literature. BMC Reprod Health.

[11] Marsh DR, Schroeder DG, Dearden KA, Sternin J, Sternin M (2004). The power of positive deviance. Br Med J.

[12] Njue C, Askew I (2004). Medicalization of female genital cutting among the Abagusii in Nyanza Province, Kenya. Frontiers in reproductive health program.

[13] Nutrition Working Group, Child Survival Collaborations and Resources Group (CORE). Positive deviance / hearth: a resource guide for sustainably rehabilitating malnourished children. Washington, DC; 2002.

[14] Population Council (2008). Female genital mutilation abandonment program – evaluation summary report.

[15] Institut National de la Statistique (INS) et ICF. Enquête démographique et de santé en Guinée 2018. Edited by ICF Ie. Conakry, Guinée, et Rockville, Maryland, United States of America; 2019.

[16] Barry AAB (2017). Guinée: l’impact des stratégies de promotion de l’abandon des mutilations génitales féminines.

[17] Gouvernement de la République de Guinée. Code de l'enfant guinéen (LOI L/2008/011/AN du 19 août 2008) – Section VII : Des violences exercées à l'encontre des enfants – Article 405 – Les mutilations génitales féminines. Conakry; 2008.

[18] Gouvernement de la République de Guinée – Ministère de la Justice (2016). Nouveau code pénal – Section II : Des mutilations génitales féminines (Articles 258–261)..

[19] Barry AAB (2019). Étude sur la perception des bénéfices que les femmes et les communautés trouvent dans la pratique des MGF.

[20] Bourdieu P (1984). Distinction: a social critique of the judgement of taste.

[21] Yoder PS, Mahy M (2001). Female genital cutting in Guinea: qualitative and quantitative research strategies. DHS analytical studies no 5.

[22] Knoblauch H (2005). Focused Ethnography. Forum: Qualitative Social Research.

[23] Triandis HC (2001). Individualism-collectivism and personality. J Pers.

[24] Van Bavel H, Coene G, Leye E (2017). Changing practices and shifting meanings of female genital cutting among the Maasai of Arusha and Manyara regions of Tanzania. Cult Health Sex.

[25] Green J, Thorogood N (2014). Qualitative methods for Health Research.

[26] Higginbottom GMA, Pillay JJ, Boadu NY (2013). Guidance on performing focused ethnographies with an emphasis on healthcare research. Qual Rep.

[27] Miles MB, Huberman AM, Saldana J (2014). Qualitative data analysis : a methods sourcebook.

[28] Maxwell JA (2010). Using numbers in qualitative research. Qual Inq.

[29] Bourdieu P, Richardson J (1986). The forms of capital. Handbook of theory and research for the sociology of education.

[30] Bourdieu P (1979). Les trois états du capital culturel. Actes de la recherche en sciences sociales.

[31] Vuarin R, Leimdorfer F, Werner JF, Gérard E, Tiékoura O. L'Afrique des individus. Itinéraires citadins dans l’Afrique contemporaine (Abidjan, Bamako, Dakar, Niamey), Édition Karthala. Paris; 1997.

[32] Kirmayer LJ, Sehdev M, Whitley R, Dandeneau SF, Isaac C. Community resilience: Models, metaphors and measures. J Aborig Health. 2009;5(1):62–117.

[33] Kirchherr J, Charles K (2018). Enhancing the sample diversity of snowball samples: recommendations from a research project on anti-dam movements in Southeast Asia. PLoS One.

[34] Gouvernement de la République de Guinée. Code de l’enfant guinéen (LOI L/2008/011/AN du 19 août 2008). Conakry; 2008.

[35] Sutton J, Austin Z (2015). Qualitative research: data collection, analysis, and management. Can J Hosp Pharm.

[36] Ritchie J, Spencer L, Bryman A, Burgess RG (1994). Qualitative data analysis for applied policy research. Analyzing qualitative data.

[37] Seale C, Silverman D (1997). Ensuring rigour in qualitative research. Eur J Pub Health.

[38] Whittemore R, Chase SK, Mandle CL (2001). Validity in qualitative research. Qual Health Res.

[39] O'Brien BC, Harris IB, Beckman TJ, Reed DA, Cook DA (2014). Standards for reporting qualitative research: a synthesis of recommendations. Acad Med.

[40] Triandis HC (1989). The self and social behavior in differing cultural contexts. Psychol Rev.

[41] Triandis HC, Gelfand MJ (1998). Converging measurement of horizontal and vertical individualism and collectivism. J Pers Soc Psychol.

[42] Bouju J, De Bruijn M. Violences structurelles et violences systémiques. La violence ordinaire des rapports sociaux en Afrique. Bulletin de l'APAD. 2008:27–8. http://journals.openedition.org/apad/3673.

[43] Mackie G (1996). Ending Footbinding and infibulation: a convention account. Am Sociol Rev.

[44] Mackie G. Female genital cutting: the beginning of the end. In: Shell-Duncan B, Hernlund Y, editors. Female "circumcision" in Africa: culture, controversy, and change. Boulder: Lynne Rienner; 2000. p. 253–81.

[45] Shell-Duncan B, Wander K, Hernlund Y, Moreau A (2011). Dynamics of change in the practice of female genital cutting in Senegambia: testing predictions of social convention theory. Soc Sci Med.

[46] Van Bavel H. At the intersection of place, gender, and ethnicity: changes in female circumcision among Kenyan Maasai. Gend Place Cult. 2019:1–22. 10.1080/0966369X.2019.1615415.

[47] Central Intelligence Agency (CIA). The World FactBook – Africa: Guinea. 2019. https://www.cia.gov/library/publications/the-world-factbook/geos/gv.html. Accessed 7 Nov 2019.

[48] Dembélé M, Oertli A, Woehrel A. Rapport de mission en Guinée du 7 au 18 novembre 2017. France: Office français de protection des réfugiés et apatrides (OFPRA) avec la participation de la Cour nationale du droit d’asile (CNDA); 2018.

[49] Mpinga EK, Macias A, Hasselgard-Rowe J, Kandala N-B, Félicien TK, Verloo H, Bukonda NKZ, Chastonay P (2016). Female genital mutilation: a systematic review of research on its economic and social impacts across four decades. Glob Health Action.

[50] Boyle E (2002). Female genital cutting: cultural conflict in the global community.

[51] Shell-Duncan B, Hernlund Y (2006). Are there "stages of change" in the practice of female genital cutting?: qualitative research findings from Senegal and the Gambia. Afr J Reprod Health.

[52] Fassin D. 1. L'ordre moral du monde – Essai d'anthropologie de l'intolérable. In: Bourdelais Patrice P, Fassin D, editors. Les constructions de l'intolérable. Études d'anthropologie et d'histoire sur les frontières de l'espace moral. La Découverte | « Recherches ». Paris; 2005. p. 17–50.

[53] Mayoux L (2001). Tackling the down side: social capital, Women’s empowerment and micro-finance in Cameroon. Dev Chang.

[54] Nega F, Mathijs E, Deckers J, Tollens E (2009). Gender, social capital and empowerment in northern Ethiopia. Munich Personal RePEc Archive.

[55] World Bank (2019). Guinea: the economic benefits of a gender inclusive society.

[56] Haut-Commissariat des Nations Unies aux droits de l’homme (2016). Rapport sur les droits humains et la pratique des mutilations génitales féminines/excision en Guinée.

[57] Bourdieu P. Choses dites. Paris: Les Éditions de minuit; 1987.

