# Correction to: Au-delà de la volonté: les conditions d’*empowerment* nécessaires pour abandonner les mutilations génitales féminines à Conakry (Guinée), une ethnographie focalisée

**DOI:** 10.1186/s12978-020-00951-6

**Published:** 2020-07-23

**Authors:** Marie-Hélène Doucet, Alexandre Delamou, Hawa Manet, Danielle Groleau

**Affiliations:** 1 Université McGill, Division de psychiatrie sociale et transculturelle, 1033 avenue Des Pins Ouest, Montréal, Québec, H3A 1A1 Canada; 2 Centre national de formation et de recherche en santé rurale de Mafèrinyah, B.P. 2649, Conakry, Guinea; 3 Université Gamal Abdel Nasser de Conakry, Conakry, Guinea; 4 Hôpital général juif, Institut Lady Davis, 4333 chemin de la Côte St-Catherine, Montréal, Québec, H3T 1E4 Canada

**Keywords:** Recherche qualitative, Ethnographie focalisée, Déviance positive, Guinée/Conakry, Mutilations génitales féminines (MGF), Capital social, Capital économique, Processus d’individualisation

**Correction to: Reprod Health (2020) 17:61**

**https://doi.org/10.1186/s12978-020-00910-1**

## Résumé simple

Les mutilations génitales féminines (MGF) peuvent entraîner des problèmes de santé immédiats et à long terme pour les filles et les femmes. De nombreuses études ont identifié les déterminants socioculturels de cette tradition, mais jusqu’à présent, dans un contexte national où les MGF sont hautement pratiquées, aucune n’a porté sur les personnes refusant de faire exciser leurs filles. La Guinée étant l’un des pays où les MGF sont les plus pratiquées dans le monde, cette recherche a exploré les profils démographiques et socioculturels de Guinéens qui ne pratiquent pas les MGF, ainsi que leur expérience de non-pratique dans un contexte de fortes prévalence et pression sociale. Nous avons constaté que les participants 1) ne divulguent pas leur statut de non-pratique de la même manière, et 2) ont des expériences variées de pression sociale. Étant donné ces expériences, nous avons surnommé les participants ainsi: 1) les “militants”, 2) les “discrets”, 3) les “courageuses”, 4) les “stratèges”. De plus, notre étude révèle qu’il ne suffit pas de *vouloir* arrêter de pratiquer les MGF. Les principales conditions permettant aux parents de ne pas faire exciser leurs filles sont: bénéficier d’un soutien social, ou être financièrement indépendants de leur réseau traditionnel de solidarité. Nous recommandons de trouver des moyens pour soutenir les femmes/familles: 1) en leur fournissant des sources de soutien social qui les aident à mettre en oeuvre décision de ne pas faire exciser leurs filles, et 2) en les aidant à acquérir une plus grande indépendance financière, notamment par la scolarisation et un meilleur accès à des emplois mieux rémunérés. Ces résultats et recommandations peuvent contribuer à améliorer ou à développer des stratégies d’intervention pour l’abandon des MGF, et donc à promouvoir la santé et le bien-être des filles/femmes.

## Contexte

Les mutilations génitales féminines (MGF) sont une modification de la zone génitale des filles/femmes, effectuée selon une logique socioculturelle en l’absence de justification médicale [1]. La pratique a le haut potentiel de provoquer des hémorragies et des infections, et d’interférer avec les processus physiologiques normaux [1], ce qui peut entraîner de graves complications pour la santé physique (ex. des problèmes urinaires, des infections, des accouchements difficiles) et même la mort [2]. Cette procédure douloureuse et souvent traumatisante [3] peut également entraîner d’importants problèmes de santé mentale (ex. trouble de stress post-traumatique, anxiété) [4]. On estime qu’au moins 200 millions de femmes et de jeunes filles actuellement en vie ont subi une MGF, et que plus de 3,6 millions de cas supplémentaires de mutilations génitales se produisent chaque année dans le monde [5, 6]. Au-delà des arguments socioculturels avancés par les sociétés pratiquantes, les MGF n’ont jamais été démontrées comme présentant des avantages pour la santé [1]. La communauté internationale considère donc que les MGF constituent une violation des droits des filles/femmes à la santé, à l’intégrité physique et à la vie (lorsque la mort résulte de la mutilation) [1, 7, 8]. Encourager et soutenir les communautés dans l’abandon de la pratique des MGF est. donc une priorité de santé publique mondiale.

Malgré une réduction globale de la prévalence des MGF, les stratégies mises en œuvre pour prévenir la pratique n’ont pas eu d’effet visible pour réduire significativement la prévalence dans certains pays [5]. Une explication potentielle est. que certaines interventions pourraient ne pas être culturellement appropriées [9]. En outre, plusieurs études (ex. des recherches qualitatives et études ethnographiques) ont identifié les discours culturels justifiant la pratique des MGF dans de nombreux pays [6], mais ne se sont pas concentrées sur les déterminants socioculturels de la *non-pratique* des MGF, le but ultime des interventions de santé publique. En outre, de nombreuses études sur les déterminants des MGF ont utilisé des devis de recherche descriptifs/de type enquêtes avec des options de réponse prédéterminées, conduisant potentiellement à des résultats limités et superficiels [2, 10]. Il est. donc nécessaire de repenser les approches et les méthodologies de recherche visant à orienter la promotion de l’abandon des MGF.

### Contexte théorique

Cette notion de “non-pratique” des MGF s’inspire du concept de “déviance positive”, qui implique que des individus dévient des normes comportementales socioculturelles en vigueur – c’est-à-dire, pour les pays à forte prévalence, des personnes qui s’écartent de l’attente de la communauté de perpétuer la pratique des MGF – et qui présentent de ce fait des caractéristiques de santé positives [11]. Dans ce contexte, le terme “déviance” n’est. pas péjoratif [12]. Les stratégies utilisant l’approche de la “déviance positive” sont basées sur la prémisse que les solutions aux problèmes qui se posent dans un contexte donné existent déjà, et que les stratégies inspirées par la communauté sont plus susceptibles d’être culturellement acceptables et réalisables, et donc durables [13, 14].

Nous avons donc cherché à appliquer le concept de “déviance positive” pour cette étude sur les MGF afin de comprendre en profondeur les dynamiques socioculturelles qui sous-tendent cette pratique, pour ultimement fournir des pistes innovantes pour informer les actions de santé publique, afin que la promotion de l’abandon des MGF soit faite d’une manière culturellement appropriée et durable.

### Population de l’étude

Pour examiner ces questions, nous nous sommes penchés sur le cas de la Guinée, où la pratique des MGF est. très répandue [15]. En fait, malgré les efforts nationaux déployés depuis plus de 30 ans pour mettre fin à la pratique [9, 16], y compris la législation anti-MGF [17, 18], les MGF persistent en Guinée avec une prévalence quasi universelle de 95 à 97% [5, 15].

La raison la plus importante pour justifier la pratique des MGF en Guinée est. le respect des coutumes ancestrales, suivie par l’objectif de restriction de la sexualité des femmes (avant et pendant le mariage), dont le but est. de sauvegarder l’honneur de la famille [9, 19]. Les “attributs positifs” des MGF semblent donc se combiner avec leur caractère traditionnel, car la pratique semble, pour beaucoup de gens, être profondément enracinée et intériorisée [9], faisant référence à la notion d’*habitus* de Bourdieu [20]. De plus, la soumission à la norme socioculturelle de l’excision des filles est. renforcée par la pression sociale, ainsi que par la stigmatisation et l’ostracisme en cas de non-respect [9]. Dans un tel. contexte, il est. virtuellement impossible d’échapper à la pratique sans subir des conséquences sociales négatives.

Bien qu’en Guinée la pratique puisse sembler à priori incontestée [21], Barry a constaté dans une étude anthropologique récente que près du tiers des Guinéens n’a pas l’intention de faire subir une MGF à leurs filles, tandis que d’autres hésitent [9]. Pourtant, on sait peu de choses sur les individus et les familles qui refusent de pratiquer les MGF en Guinée. Quantitativement, on sait que la prévalence de la pratique des MGF varie légèrement en fonction de facteurs sociodémographiques. Par exemple, les chrétiens pratiquent moins que les musulmans (77,9% contre 97,1%) [15]. Des variations existent également entre les groupes ethniques: ceux qui pratiquent le moins sont les Toma (69,3%), les Guerzé (77,8%) et les Kissi (88,2%), par opposition aux Soussou (97,9%), aux Peul (97,3%) et aux Malinké (95,9%) [15]. Cependant, selon les dernières données, la prévalence des MGF dans les zones urbaines (94,8%) est. comparable à celle des zones rurales (94,3%), avec la capitale Conakry affichant une prévalence de 95,6% [15]. Qualitativement, l’étude de Barry indique que les familles qui ne pratiquent pas les MGF ne dissimulent pas leur statut, mais ne publicisent pas non plus leur décision et restent relativement invisibles pour le reste de la population [9]. On ne connaît toutefois pas leurs croyances, leurs motivations et leurs justifications pour aller à l’encontre des normes socioculturelles, le contexte dans lequel elles vivent et leur expérience relativement à la pression sociale.

Il est. donc nécessaire de comprendre les déterminants socioculturels de la *non-pratique* des MGF dans le contexte guinéen de fortes prévalence et pression sociale, afin de proposer des stratégies culturellement appropriées qui permettraient de soutenir les familles souhaitant abandonner la pratique. Pour combler ce manque de connaissances, les questions de recherche qui ont guidé cette étude étaient les suivantes: 1) Quel est. le profil démographique et socioculturel de membres de familles qui ne pratiquent pas les MGF en Guinée? 2) Quelle est. leur expérience de non-pratique des MGF? 3) Comment font-ils face à la pression sociale qui incite à la pratique des MGF? 4) Quelles conditions leur permettent de mettre en oeuvre leur décision de ne pas pratiquer les MGF, et ainsi d’agir contre la norme socioculturelle en vigueur?

## Méthodes

### Méthodologie

Les comportements en matière de santé sont souvent complexes et profondément ancrés dans les contextes socioculturels. Afin d’acquérir une compréhension approfondie de la logique socioculturelle qui sous-tend la non-pratique des MGF en Guinée, nous avons utilisé la méthodologie de “l’ethnographie focalisée” [22]. En fait, étant donné que les dynamiques socioculturelles liées à la pratique des MGF sont hétérogènes en fonction de divers facteurs (ex. les régions géographiques) [6] et évoluent dans le temps [23, 24], nous soutenons qu’il faudrait une mobilisation colossale de ressources (y compris de temps) pour réaliser une ethnographie *classique* avant de mettre en œuvre chaque stratégie de santé publique afin qu’elle soit adaptée à son contexte socioculturel [9], puisque ce type d’exploration implique une longue immersion sur le terrain [25]. L’ethnographie *focalisée* est. donc une approche plus pragmatique puisque “ce type particulier d’ethnographie sociologique” [22] combine: 1) la connaissance de la communauté à l’étude par une recension de la littérature, et par l’association avec des chercheurs locaux (AD et HM dans la présente étude) agissant notamment à titre de courtiers culturels; 2) des visites de terrain plus courtes ainsi que l’utilisation d’outils de recherche qualitative (ex. entretiens focalisés/semi-structurés); et 3) une analyse rigoureuse du matériel recueilli [26]. Du reste, bien que ces analyses aient inclus l’utilisation de nombres tels que des fréquences simples pour détecter des *patterns* dans les données [27, 28], cet article présente un sous-ensemble d’une approche plus large d’ethnographie focalisée.

### Cadre conceptuel

Nous avons utilisé trois types de concepts pour explorer les conditions et les formes de pouvoir nécessaires pour mettre en œuvre la décision de ne pas pratiquer les MGF: 1) le capital total; 2) l’interdépendance vs l’individualisme; et 3) la résilience. Le concept de “capital” fait référence aux avantages objectifs ou subjectifs auxquels les individus peuvent avoir accès dans leur environnement social, leur permettant d’acquérir du pouvoir; le **capital total** est. l’accumulation de diverses sources de pouvoir qui peuvent varier en fonction du contexte [29]. Cette notion comprend les formes culturelle, sociale et économique du capital. Le **capital culturel** fait référence à l’acquisition d’avantages liés à la culture; cela peut faire référence à l’éducation, aux connaissances et aux compétences intellectuelles, ou prendre la forme d’objets ayant une valeur culturelle. Plus précisément, nous avons considéré le capital culturel *institutionnalisé*, qui consiste en l’acquisition de pouvoir par le biais de qualifications académiques [30]. Pour cette étude, nous avons donc utilisé le niveau d’éducation des participants afin de déterminer leur degré d’accès au capital culturel institutionnalisé (que nous appelons par la suite “capital culturel” par souci de concision). Le **capital social** consiste à avoir accès à des réseaux sociaux au sein desquels un soutien est. apporté au quotidien et dans les moments difficiles; ce système de solidarité implique également de respecter et de soutenir les décisions de tout un chacun. Pour cette étude, nous avons cherché à savoir si les participants bénéficiaient d’un soutien social pour ne pas pratiquer les MGF (référé comme étant un “capital social positif”), ou s’ils rencontraient des problèmes sociaux étant donné leur décision de ne pas pratiquer les MGF (référé comme une absence de capital social). Le **capital économique** fait référence au pouvoir financier, et donc à l’indépendance correspondante, laquelle permet aux individus de prendre leurs propres décisions de vie. Pour cette étude, nous avons utilisé les indicateurs de possession de biens et d’accès à des services de base,^1^ et de taux d’occupation du logement des ménages,^2^ comme proxy pour évaluer le niveau économique de la famille des participants.^3^ L’**interdépendance** est. définie comme un système “traditionnel” de solidarité familiale ou clanique, qui implique une forme de protection sociale et, dans certains cas, l’accès à des sources de financement; cette connexion à d’autres implique souvent des attentes en matière de lignage [31]. En revanche, l’**individualisme** peut être défini comme l’émancipation de la personne des valeurs de sa famille ou de sa communauté, et comme l’indépendance et l’autonomie de la personne, qui peut évoluer dans des champs sociaux séparés et hétérogènes [31]. Enfin, la composante psychologique de la **résilience** consiste en la capacité à réguler ses émotions tout en faisant face à l’adversité, à s’adapter et à atteindre le bien-être [32].

### Échantillonnage et recrutement des participants

Le recrutement a eu lieu à Conakry, la capitale de la Guinée, car nous avons anticipé que davantage de “déviants positifs” potentiels pourraient être joints en un temps limité dans un tel. milieu urbain, maximisant ainsi la faisabilité de notre étude. Nous avons recruté les participants en utilisant la méthode de l’échantillonnage “boule de neige” [27], en demandant à des organisations locales qui font la promotion de l’abandon des MGF, à des membres de la communauté (via des conversations informelles), et éventuellement à des participants de l’étude, de nous diriger vers des participants potentiels. Cette méthode s’est. avérée efficace, car les “déviants positifs” appartiennent à une sous-population rare et cachée qui peut être difficile à identifier [33]. Nous avons veillé à ce que l’échantillon soit de “variation maximale” [27], c’est-à-dire que les participants ont été sélectionnés autant que possible en fonction d’une hétérogénéité de facteurs, à savoir selon la génération (c.-à-d. des jeunes adultes célibataires (18–30 ans); des parents (incluant des belles-mères/coépouses ou des tantes); et des grands-parents), ainsi que selon les variables sociodémographiques (c.-à-d. le niveau d’éducation, l’ethnicité, etc.). Comme en Guinée les MGF sont généralement pratiquées entre la naissance et l’âge de 15 ans [15], les participants pouvaient être inclus dans notre étude s’ils appartenaient à des familles ayant au moins une fille non-excisée de 16 ans ou plus, ou des filles de moins de 15 ans avec la ferme affirmation parentale qu’elles ne seront pas excisées. Les jeunes adultes qui n’étaient pas encore parents pouvaient participer s’ils exprimaient une conviction claire contre la pratique. Notre échantillon pouvait comprendre des participantes qui ont elles-mêmes été excisées. Les mineurs (âgés de moins de 18 ans [34]) n’ont pas été invités à participer, mais aucun autre critère d’exclusion n’a été appliqué dans le recrutement des participants/familles. Compte tenu de notre budget limité, le recrutement des participants a pris fin après l’inclusion de 30 personnes.

### Collecte de données

Comme les discussions individuelles sont généralement plus appropriées pour explorer des sujets sensibles tels que les MGF [25], des entretiens individuels semi-structurés et approfondis ont été menés. Le guide d’entretien comprenait les sections suivantes: 1) une partie narrative (non structurée), pour permettre aux participants de s’exprimer librement sur leur expérience personnelle de non-pratique des MGF dans le contexte socioculturel de Conakry; 2) les incidences d’appartenir à une famille non-pratiquante sur le capital social des participants; 3) l’histoire de la résilience des participants qui vivent des problèmes étant donné leur statut de non-pratique; et 4) des données sociodémographiques, soit leurs: âge; niveau d’éducation; religion; identité ethnique; occupation; et le niveau économique de leur famille.

Les entretiens se sont déroulés dans des espaces privés, à la convenance des participants, afin qu’ils puissent s’exprimer librement sans être entendus par leur famille ou leur entourage. Les discussions se sont déroulées en français ou dans la langue avec laquelle les participants se sentaient les plus à l’aise, et ont duré en moyenne 47 min. Tous les entretiens ont été enregistrés numériquement. La collecte de données a été effectuée au cours des mois de janvier et février 2019. Les verbatim en français ont été littéralement transcrits par un transcripteur professionnel indépendant, et les entretiens menés dans les langues locales (soussou, malinké, pular) ont été transcrits en français par HM. Toutes les données transférées via le Web ont été envoyées par transfert digital encrypté, afin d’assurer la sécurité du matériel recueilli [35].

### Analyse des données

Nous avons utilisé la méthode du *“Framework analysis”* pour analyser les données, car cette approche: facilite la vue d’ensemble de chaque cas; permet des comparaisons et des contrastes entre les expériences, les points de vue et les situations de vie des participants; et aide à identifier les thèmes-clés, ainsi qu’à révéler les *patterns* et les typologies dans les données [27, 36]. Cette méthode a consisté en cinq étapes: 1) “se familiariser avec les données”, en écoutant les fichiers audio et en lisant les transcriptions; 2) “identifier un cadre thématique”, à partir des thèmes qui ont émergé des données; 3) “indexer”: coder tous les verbatim de manière systématique à l’aide du logiciel MAXQDA; 4) “créer une matrice”: 4. 1) produire des résumés pour chaque cas en fonction des thèmes principaux, et 4.2) exposer tous les cas dans une matrice; 5) “schématiser et interpréter”: procéder à 5.1) une analyse intra-cas, et 5.2) une analyse inter-cas, en comparant et en contrastant tous les cas pour chaque thème afin de révéler les typologies et les *patterns* dans les données. Cette dernière étape a également impliqué une analyse critique afin de tirer des recommandations pour informer les politiques et les stratégies. Cette interprétation des résultats s’est. appuyée sur des connaissances théoriques ainsi que sur les expériences et les observations des chercheurs relativement au contexte socioculturel.

### Réflexivité et positionnalité

Alors qu’elles informaient les participants potentiels sur les objectifs de l’étude, les intervieweuses (MHD et HM) ont précisé que le but ultime de l’étude était de faire des recommandations, sur la base de leurs récits, pour les stratégies de promotion de l’abandon des MGF en Guinée. Nous ne pensons toutefois pas que les réponses des participants aient été particulièrement biaisées (désirabilité sociale) à cause de cette déclaration, étant donné que plusieurs d’entre eux étaient déjà actifs dans la lutte contre les MGF dans leur réalité quotidienne, et que les réponses des autres étaient cohérentes avec celles des militants.

De plus, MHD étant une femme occidentale blanche s’est. positionnée comme une “étrangère”. Cela a légitimé le fait de demander aux participants d’expliciter leurs expériences et leurs points de vue, car ils auraient pu être tentés d’insinuer des significations culturelles s’ils avaient uniquement parlé à un intervieweur guinéen, en raison des tabous entourant les MGF ou des connaissances locales prises pour acquises [25]. De façon complémentaire, HM étant Guinéenne, était une “initiée” [dans le sens de “*insider*”, qui vient de ce milieu] qui a facilité la prise de contact avec les participants potentiels et l’établissement d’une relation de confiance [25]; elle a également mené les entretiens qui ont été réalisés dans les langues locales. Cette “mixité” de positionnalités a donc été un atout qui a permis d’approfondir les données recueillies.

### Fiabilité

Différentes mesures ont été utilisées pour assurer la crédibilité des résultats. La partie narrative des entretiens a commencé par la question: “Quand avez-vous entendu parler des MGF pour la première fois? ”, permettant aux participants de se plonger dans l’histoire de leur propre expérience de manière plus naturelle et chronologique, et de partager l’évolution de leurs réflexions. L’exactitude des transcriptions a été vérifiée par MHD; l’exactitude de la traduction et de la signification socioculturelle des entretiens traduits a été vérifiée [25] par un assistant de recherche indépendant, puis par MHD. Pendant la phase de “création de la matrice”, MHD a saisi les données codées dans la matrice et a effectué des contrôles de qualité avec les données brutes [27]; HM a confirmé l’adéquation procédurale des données exposées dans la matrice [27]. La technique du “simple comptage” a permis de confirmer les *patterns* trouvés dans les données, par opposition aux observations anecdotiques [37]. L’interprétation des résultats a d’abord été effectuée par MHD, et a par la suite été validée de plusieurs manières, par: la vérification par les co-chercheurs guinéens agissant en tant que “courtiers culturels” (AD, HM); la “vérification par des experts” [38]; la vérification dans la littérature de la congruence avec des phénomènes similaires [27]; l’analyse de la plausibilité des résultats [27]; et l’inclusion de données brutes dans notre article scientifique pour appuyer les résultats et les interprétations [25].

### Transférabilité

Étant donné que les résultats ont été validés relativement à leur adéquation culturelle, nous sommes d’avis que nos recommandations sont transférables aux Guinéens qui souhaitent abandonner la pratique des MGF et qui vivent à Conakry, ainsi que dans d’autres milieux urbains guinéens [25, 27]. En outre, nous pensons que nos conclusions et recommandations pourraient être applicables à d’autres Guinéens et Ouest-Africains qui souhaitent abandonner les MGF, mais nous recommandons de tester et de vérifier cette présupposition [25, 27].

### Rapport

Nous avons utilisé les lignes directrices pour l’établissement de rapports de la SRQR [39].

### Considérations éthiques

Cette étude a reçu l’approbation éthique du Comité d’éthique de la recherche de l’Université McGill ainsi que du Comité national d’éthique pour la recherche en santé de la République de Guinée. Les chercheurs ont obtenu le consentement libre de la part des participants après leur avoir expliqué et fait lire le formulaire de consentement détaillé. En outre, les participants ont tous été invités à donner leur accord avant l’enregistrement des entretiens. Aucun mineur n’a été interviewé. Tous les entretiens ont été anonymisés (un code a été attribué aux entretiens). Enfin, les données sont stockées dans l’ordinateur de MHD, lequel est. protégé par un mot de passe.

## Résultats

Dix-huit femmes et douze hommes ont accepté de participer à notre étude, pour un total de 30 personnes âgées de 18 à 69 ans et représentant trois générations: jeunes adultes (18–30 ans), parents et grands-parents (Tableau 1); aucun “jeune adulte” n’avait d’enfant, à l’exception d’une femme célibataire. Les participants étaient généralement enthousiastes à l’idée de participer, et certains se sont même portés volontaires pour être interviewés sans être sollicités, ayant entendu parler de notre étude par des membres de leur réseau. Trois personnes ont refusé de participer, craignant: que l’interview soit diffusée à la radio; d’être accusées de “suivre les Blancs” par la population environnante; et en tant qu’imam, qu’il soit jugé par ses fidèles. Ces refus sont survenus en dépit que nous ayons expliqué que les entretiens auraient lieu dans un endroit privé de leur choix et seraient anonymes, et que les enregistrements audio ne seraient utilisés que pour les besoins de l’étude.

### Profils démographiques et socioculturels

Notre échantillon était principalement composé de personnes très instruites, avec près de la moitié d’entre elles (14/30) ayant un diplôme universitaire (Tableau 1). La plupart des participants étaient musulmans (24/30). Les personnes interrogées représentaient un éventail relativement large de groupes ethniques, mais près d’un tiers d’entre elles étaient des Malinkés (9/30). Bien que tous les participants partageaient la conviction que les MGF sont nocives, seulement un d’entre eux – un jeune homme natif d’une région sénégalaise où les MGF ne sont pas pratiquées – a déclaré être issu d’une famille qui ne pratique pas les MGF depuis de nombreuses générations. Cela confirme que virtuellement tous les participants étaient issus d’une culture où les MGF sont la norme. Seules 3/18 femmes n’avaient pas subi de MGF elles-mêmes. Hormis le jeune homme mentionné ci-dessus, tous les hommes interrogés (11/12) ont mentionné être mariés à une femme excisée ou avoir des sœurs excisées.

Tableau 1 Caractéristiques démographiques des participants (*n* = 30)

**Table:** 

		n	Pourcentage (%)
Génération	Grands-parents	4	13
	Parents	18	60
	Jeunes adultes	8	27
Sexe	Femmes	18	60
	Hommes	12	40
Niveau d’éducation	Université – doctorat	1	3
	Université – maîtriser	8	27
	Université – baccalauréat	5	17
	*Actuellement aux études (niveau secondaire/collégial)*	*5*	*17*
	Secondaire ou formation professionnelle	6	20
	Primaire or aucune éducation	5	17
Religion	Musulmans	24	80
	Chrétiens	6	20
Identité ethnique	Malinké	9	30
	Peul	6	20
	Badiaranké	5	17
	Soussou	4	13
	Kissi	4	13
	Guerzé	1	3
	Manon	1	3

### Expérience de la non-pratique des MGF et de la pression sociale

Premièrement, les participants ont démontré des *patterns* distinctifs concernant leurs attitudes et comportements relativement au degré auquel ils divulguent (ou non) leur statut de non-pratique dans leur vie quotidienne. Certaines personnes ont déclaré assumer pleinement leur décision de ne pas faire exciser leurs filles, et promeuvent même l’abandon de l’excision des filles. D’autres ont mentionné qu’elles ne font pas la publicité de leur statut de non-pratique, mais ne le cachent pas à tout prix non plus. Et certains ont confié qu’ils cachent non seulement leur statut, ils utilisent la “tromperie”, en d’autres mots, ils mentent à ceux qu’ils considèrent comme menaçants en faisant semblant de faire exciser leurs filles. Deuxièmement, il a été constaté que la perception des participants d’être soumis (ou non) à une pression sociale pour faire exciser leurs filles était variée, et même polarisée. En effet, certains ont dit ne pas être soumis à la pression ou ont déclaré ne pas être affectés par les attentes de la société. D’autres ont révélé subir de lourdes conséquences sociales pour ne pas se conformer à la norme. Les autres ont expliqué comment ils ont réussi à éviter les troubles sociaux: soit en étant discrets, soit en utilisant la “tromperie”. Une typologie a donc été créée pour décrire les participants en fonction de leurs différents profils et expériences, que nous avons surnommés ainsi: 1) les “militants”, 2) les “discrets”, 3) les “courageuses”, 4) les “stratèges” (voir Figure 1). Les points communs et les singularités de leurs capitaux culturel, social et économique sont présentés au Tableau 2^4^; la description de leurs expériences suit.

**Fig. 1:**
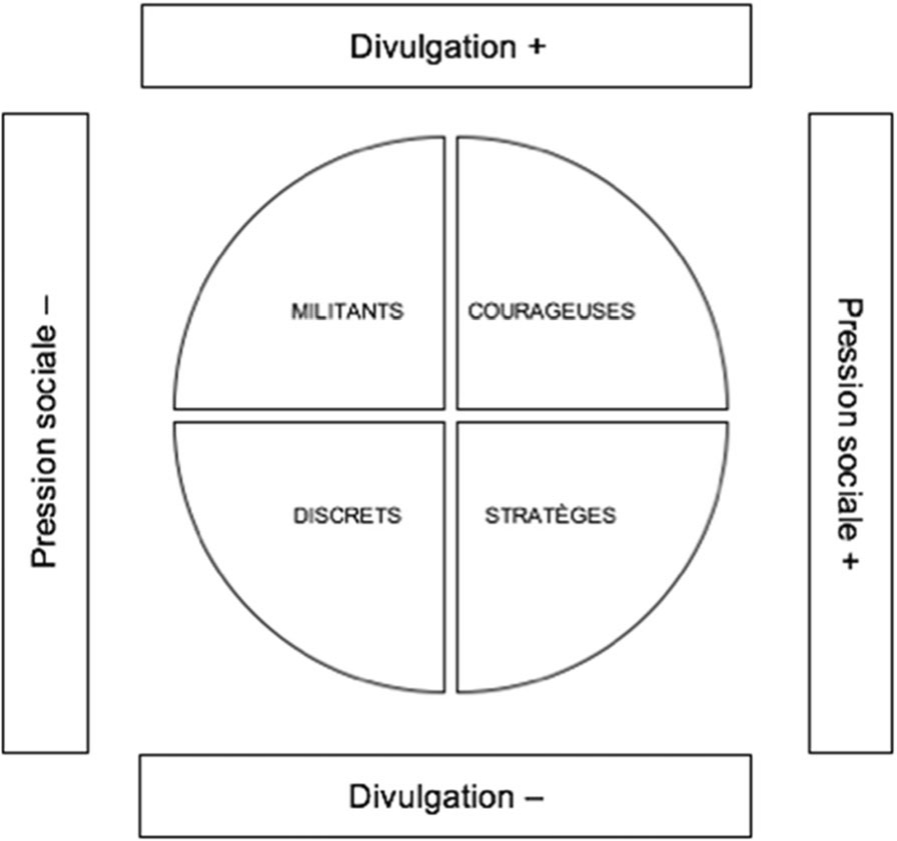
Niveau de divulgation de la non-pratique des MGF vs expérience de pression sociale

Les “militants” disent ouvertement qu’ils sont opposés aux MGF, et ne craignent/perçoivent pas la pression sociale.

Les “discrets” évitent les troubles sociaux en ne divulguant pas leur décision, mais ne la cachent pas non plus à tout prix.

Les “courageuses” disent ouvertement qu’elles sont opposées aux MGF, et souffrent des conséquences sociales correspondantes.

Les “stratèges” évitent les troubles sociaux en trompant/mentant à propos de leur décision.

Tableau 2 Résumé des caractéristiques des participants selon la typologie (*n* = 30).

**Table:** 

		Militants	Discrets	Courageuses	Stratèges	Total
n		14	12	2	2	30
Capital social^5^	Oui	14	12	0		26
	Non	0	0	2	2 (partiel)^6^	4
Capital culturel	Université – doctorat	1	0	0	0	1
	Université – maîtrise	4	4	0	0	8
	Université – baccalauréat	2	3	0	0	5
	*Actuellement aux études*	4	1	0	0	5
	Secondaire ou formation professionnelle^7^	1	2	1	2	6
	Primaire or aucune éducation	2	2	1	0	5
Capital économique	Élevé	10	9	1	1	21
	Moyen	4	3	1	1	9
	Faible	0	0	0	0	0
Type de liens sociaux	Discours individualiste	13	7	0	0	20
	Discours d’interdépendance	0	0	2	1	3
	*Données manquantes*^8^	*1*	*5*		*1*	*7*

**Les “militants”.** Les quatorze participants classés comme “militants” ont affirmé qu’ils parlent ouvertement de leur opposition à la pratique des MGF et, pour ceux qui sont parents, de ne pas faire exciser leurs propres filles. Militer contre les MGF fait partie de leur travail, ou est. quelque chose qu’ils font auprès de personnes de leur entourage.

*« J’ai décidé de combattre [la pratique des MGF]. La question pour moi ce n’est plus est-ce que je vais la pratiquer, non, ça, c’est révolu; mais c’est pour combattre la pratique »* (père, niveau de maîtrise, travaille comme militant dans une ONG).

Ils ont également tous confirmé ne pas avoir de problèmes liés à la pression sociale, et vivre dans un contexte social favorable leur permettant de mettre en œuvre leur décision de ne pas faire exciser leurs filles. De plus, ils ne craignaient pas d’être critiqués.

*« Avant, [ce que les gens disaient du fait que je n’étais pas excisée] me dérangeait, parce que quand tu es enfant et que tu [es] dans un milieu où tu es avec des amies et que c’est toi qui es [critiquée], c’est dérangeant. Mais aujourd’hui […] je suis fière [de ne pas être excisée]* (jeune femme non-excisée, niveau de baccalauréat).

Ce sous-échantillon comprenait de nombreuses personnes ayant un niveau d’éducation et un capital économique élevés (Tableau 2). Leurs occupations étaient variées: ils étaient étudiants, gestionnaires d’ONG, experts juridiques (avocats et juristes), professionnelle de la santé (sage-femme), enseignant et commerçants (caissière et vendeuse). En outre, tous ont exprimé des valeurs individualistes dans leur récit, telles que:

*« Ce sont mes enfants, je n’ai pas à recevoir d’ordres de qui que ce soit »* (père, niveau de maîtrise).

*« Je ne vais jamais écouter [ce que les gens disent] jusqu’à sacrifier mon enfant. Si tu veux, continue à te moquer de moi. Mais je ne t’écouterai pas pour que tu tues mon enfant »* (mère, aucune éducation).

Certains parents ont dit qu’ils devaient protéger leurs filles de certains membres de leur famille – généralement des tantes et des grand-mères paternelles – qui pourraient potentiellement “voler” leurs enfants pour les faire exciser dans leur dos.^9^

**Les “discrets”.** Douze participants ont mentionné ne pas parler ouvertement de leur décision de ne pas faire exciser leurs filles, sauf à un groupe de personnes (famille immédiate ou amis) avec lesquelles ils partagent des valeurs communes.

*« Cette décision, je l’ai pas rendue publique. On est en Afrique, chacun qui a sa manière de penser. Moi, ma décision est que je [ne] veux pas exciser mes filles. Ça s’arrête là » (père, niveau de maîtrise).*

Cependant, ils ne tentent pas activement de cacher leur statut à tout prix. Ils ont également tous affirmé n’avoir aucun problème social concernant leur décision, ou ont dit ne pas être sensibles aux influences sociales.

*« Certains peuvent dire ce qu’ils en pensent, mais ça ne me dérange pas […] parce que je peux les convaincre pourquoi j’ai décidé, mais eux, ils [ne] peuvent pas me convaincre pourquoi ils le font. […] On est tranquilles [avec notre décision] »* (grand-père & père de filles non-excisées, niveau de maîtrise).

Très peu de personnes interrogées ont explicitement mentionné que leur discrétion de ne pas faire connaître leur point de vue avait pour but d’éviter les problèmes sociaux.

*« Je conseillais les femmes, je leur disais d’arrêter [de pratiquer les MGF]. Mais à chaque fois que tu abordes ce sujet, s’il y a les vieilles femmes, elles vont te regarder comme si tu es une personne ignorante, satanée, contre nos coutumes, déracinée, perdue,… Tu vois… Donc finalement [je] n’en parle plus »* (tante, niveau de maîtrise).

Certains “discrets” ont également fait allusion au phénomène du “vol” des filles. Certaines femmes ont dit avoir été “volées” à leur mère pour être excisées lorsqu’elles étaient enfants. D’autres ont averti leur famille élargie de ne pas prendre leurs filles pour les faire exciser, sinon ils les dénonceraient aux autorités judiciaires.

D’autres participants ont exprimé que de nos jours à Conakry, une proportion croissante de personnes ne s’intéresse pas au sujet des MGF et n’en discute plus, comme si pour certains c’était un problème résolu d’une époque révolue, ou si c’était devenu davantage une affaire privée.

*« Personne n’en parle de cette pratique. […] On ne me demande pas [si j’ai été excisée]. On ne m’a jamais demandé ça »* (jeune femme non-excisée, niveau de baccalauréat).

Une proportion importante de ce sous-groupe comprenait des personnes ayant un niveau d’éducation élevé ou très élevé, et pour la plupart un capital économique élevé (Tableau 2). Ils étaient soit ingénieurs (4/12), professionnels de la santé, travailleurs dans l’industrie du commerce (ex. caissière, coiffeuse), femmes de ménage ou étudiants. Un participant était à la recherche d’un emploi. Deux personnes âgées étaient à la retraite. En outre, la plupart de leurs récits reflétaient des valeurs individualistes.

*« C’est toi-même qui dois prendre une décision pour dire que ta fille ne sera pas excisée parce que c’est ta fille, personne d’autre ne doit te forcer. Moi en tout cas, j’ai décidé que mes filles ne seront pas excisées, c’est ma décision »* (mère, niveau d’éducation primaire).

**Les “courageuses”.** Deux personnes interrogées – une mère et une grand-mère – ont révélé que dès que les gens autour d’elles ont su que leurs filles ne seraient pas excisées, elles ont été critiquées et rejetées. Cela leur a causé beaucoup de souffrances, car elles vivaient dans un environnement social hautement hostile à ceux qui osent ne pas se conformer à la tradition des MGF.

*« Un jour, ma belle-famille m’a dit d’envoyer ma première fille pour la faire exciser, j’ai refusé. Elles m’ont insultée; [Quand] mes filles approchent [de mes voisines], elles disent*: *« quittez à côté de nous, celles qui ne sont pas excisées n’ont qu’à aller de l’autre côté. Si une personne n’est pas excisée, c’est un animal »* (mère, niveau d’éducation primaire).

*« Eeeeh !! Elles étaient fâchées contre moi !! On m’appelait même « la folle ». [Elles disaient*:*] « Elle, elle n’a pas besoin de vivre, elle va voir. » […] Je n’allais chez personne, personne ne venait chez moi […] Je me sentais… frustrée, je me sentais isolée »* (grand-mère, école professionnelle).

La mère a surtout insisté sur le fait qu’elle devait constamment garder un œil sur ses filles afin qu’elles ne soient pas “volées” par des membres de la famille pour être excisées. Elles ont exprimé leur volonté d’être braves en dépit de l’adversité, en s’appuyant sur leur foi et, surtout, en se sentant en paix à l’idée de faire ce qu’elles estiment être le mieux pour leurs filles, faisant ainsi preuve d’un haut niveau de résilience.

*« J’ai même divorcé avec mon mari, tellement que j’avais de la pression sociale. Mais ! Je suis restée dure. On me disait que je suis une femme trop dure. Ah ! ah ! Ce que je fais, c’est pour l’avenir des enfants »* (grand-mère, école professionnelle).

De plus, non seulement elles assument ouvertement leur décision de ne pas faire exciser leurs filles en dépit du lourd coût social qu’elles doivent endurer, mais elles ont même témoigné qu’elles plaident pour la non-pratique des MGF: *“Si je vois quelqu’un qui le fait, je lui conseillerai d’arrêter en lui disant les conséquences”* (mère, niveau primaire). L’aînée a même fondé une association pour sauver les filles de l’excision à une époque où la pression sociale était encore plus intense qu’aujourd’hui. Nous les avons donc classées comme étant “courageuses”.

De plus, la mère a spontanément confié qu’en plus d’être mise à l’écart par son lignage, elle a été abandonnée par son mari pendant cinq ans en raison de sa décision d’éviter les MGF pour leurs filles; elle a donc dû prendre en charge toutes les dépenses pour ses filles et pour elle-même. Malgré son capital économique moyen et ses soucis financiers, ses revenus de commerçante (restaurant) étaient suffisants pour lui permettre d’être complètement indépendante économiquement. La dame âgée était une sage-femme à la retraite avec un capital économique élevé. Aucune de ces participantes n’a exprimé de valeurs individualistes.

**Les “stratèges”.** Deux mères ont adopté la stratégie consistant à dissimuler la vérité sur leur décision de ne pas faire exciser leurs filles, car elles pensaient qu’elles seraient autrement confrontées à des problèmes importants émergeant de leur environnement social.

*« Quand on dit de faire exciser ta fille, tu réponds que […] sa tante paternelle s’en occupera. […] Tu l’envoies au centre de santé, et puis tu reviens dire au quartier qu’elle a été excisée*: *qui te dira de lui écarter les jambes pour vérifier ? Personne »* (mère, niveau d’éducation secondaire).

*« Ils vont m’obliger de le faire. Je vais […] tromper l’apparence, mais je ne ferais pas la pratique. […] Je ne le dirai pas, même à mon mari je ne le dirai pas. […] [C’est] la méthode la plus facile pour éviter des problèmes, mais tout en arrêtant quand même, en abandonnant l’excision »* (mère, école professionnelle).

Les motivations qui sous-tendent leur recours à la “tromperie” sont multiples. Tout d’abord, elles ont expliqué qu’en plus d’épargner à leurs filles d’être excisées, elles agissent ainsi afin qu’elles ne se fassent pas “voler” par un membre de leur famille pour être excisées à leur insu. Deuxièmement, elles protègent leurs filles non-excisées d’être stigmatisées et ostracisées par leurs paires – protégeant ainsi le statut social des filles. Troisièmement, les mères agissent de cette manière pour s’épargner la pression sociale incitant à se conformer à la tradition. Enfin, bien qu’elles aient toutes deux dit bénéficier d’un soutien pour leur décision au sein d’un cercle social très restreint, elles ont dû recourir à la tromperie avec certaines personnes pour protéger leur propre capital social (ex. pour protéger leur mariage).

Ces femmes avaient un niveau d’éducation moyen et vivaient dans des contextes socio-économiques élevés (Tableau 2); mais bien que la femme qui cache la vérité à son mari travaille dans l’industrie du commerce, son récit ne nous permet pas de savoir si elle pourrait être financièrement indépendante en cas de divorce. L’autre femme était femme de ménage. Aucune de ces participantes n’a exprimé de valeurs individualistes, ce qui laisse supposer qu’elles vivent dans un contexte d’interdépendance.

Ce stratagème est. mis en œuvre de différentes manières, comme le montrent les exemples suivants. Certaines amènent leurs filles au centre de santé pour demander au professionnel de la santé de faire semblant de pratiquer une excision, ou de faire une légère incision sur la région génitale de la fille pour qu’elle croie qu’elle a été excisée: de cette façon, les mères s’assurent que leurs filles ne révèlent pas qu’elles ne sont pas excisées, évitant ainsi qu’elles soient “volées” pour subir l’intervention. D’autres font porter à leurs filles les vêtements traditionnels post-excision (un pagne coloré noué sur la poitrine), pour “tromper l’apparence”.

Bien que très peu de personnes interrogées ont déclaré utiliser la “tromperie”, de nombreux participants et personnes rencontrés de manière informelle ont fait allusion à cette stratégie. Parmi eux, une grand-mère que nous avons classée comme “discrète” – puisqu’elle est. actuellement discrète au sujet du statut de non-excision de ses petites-filles – a expliqué qu’elle a utilisé la stratégie de la tromperie pour épargner l’excision à sa fille cadette. Cette stratégie semble être utilisée de façon transitoire, jusqu’à ce que la norme sociale apparente montre une tendance à la non-pratique des MGF.

## Discussion

Nos données confirment que la culture guinéenne est. apparemment favorable à la pratique des MGF et que la pression sociale en faveur de la perpétuation des MGF persiste – comme le montrent l’expérience des “courageuses” et le besoin des “stratèges” d’utiliser la tromperie pour éviter de souffrir d’une telle pression – ce qui se produit fort probablement au sein des strates les moins éduquées et privilégiées de la population. Elles confirment également l’existence d’un système de punition, sous la forme de stigmatisation et d’exclusion, visant à promouvoir la conformité à la tradition des MGF. Certaines personnes peuvent en effet ne pas se sentir capables de faire face au coût social élevé d’être stigmatisées et ostracisées en cas d’environnement social hostile, et cèdent donc et font exciser leurs filles. Ces constats correspondent à la description des modes de fonctionnement des sociétés collectivistes traditionnelles, lesquelles sont caractérisées par la subordination des intérêts personnels des individus au profit du bien-être des familles/collectivités [40, 41]. Dans un tel. contexte, les personnes appartenant à des catégories sociales considérées comme étant inférieures – généralement les femmes et les enfants – souffrent souvent de diverses formes de “violence ordinaire” [42], comme les MGF. Nos constats sont également en phase avec la théorie des normes sociales et en particulier la théorie des conventions sociales, laquelle postule que les actions individuelles sont conditionnées par des attentes interdépendantes [43–45].

Pourtant, en écho à l’étude de Barry [9], nous aussi avons facilement trouvé des personnes ne pratiquant pas les MGF (qui étaient même enthousiastes à participer à notre étude), et nous soutenons que cela pourrait indiquer que des changements commencent à se produire dans la société guinéenne en faveur de l’abandon des MGF, du moins dans le contexte urbain de Conakry. En effet, comme les sociétés rurales sont généralement caractérisées par un nombre limité d’options de groupes sociaux, il est. habituellement plus facile de “contrôler” les membres de la communauté et de préserver les traditions [40]. En revanche, les environnements urbains sont caractérisés par une plus grande diversité de cultures, ce qui facilite les processus d’individualisation et permet aux gens de prendre des distances par rapport aux pratiques traditionnelles perpétuées à travers les relations sociales [31, 40]. Ce clivage urbain/rural est. documenté pour la pratique des MGF, qui est. généralement moins répandue dans les villes que dans les zones rurales [6]. Même si cette tendance n’est. pas statistiquement observable en Guinée [15], il est. probable que le fait de vivre dans la capitale guinéenne offre des opportunités à ses habitants pour abandonner la pratique des MGF. Mais hormis le simple fait de vivre dans un contexte urbain, des conditions favorables – telles que la scolarisation et l’autonomie financière – sont généralement requises pour s’émanciper des contraintes de la dépendance socioculturelle [31] lorsque le changement collectif ne semble pas être envisageable et qu’il doit donc être fait sur une base individuelle.

En outre, un résultat inattendu et important de notre étude est. que la plupart des personnes interviewées (les “militants” et les “discrets”) ont affirmé ne pas éprouver de problème significatif lié à la pression sociale pro-MGF. Le travail très récent de Barry (2019) montre en effet que de nos jours, près de la moitié des femmes vivant à Conakry (46%) perçoivent qu’aucune sanction sociale ne résulte de la non-conformité à la tradition des MGF, tandis qu’une proportion moindre (29%) considère que la pression sociale existe toujours [19], ce qui laisse présager une diminution de la pression sociale. Cependant, les conditions qui ont favorisé ce changement n’ont pas été examinées. Cela pourrait être dû à l’hypothèse susmentionnée selon laquelle les attitudes changent progressivement en Guinée relativement aux MGF. Mais en dépit de cette tendance émergente, et malgré le fait que de nombreux Guinéens préféreraient ne pas faire exciser leurs filles [9], la prévalence des MGF reste élevée même chez les plus jeunes générations de filles guinéennes, puisque 92% des 15–19 ans sont excisées [15]. Toutefois, la mise en lumière de la tendance de la “tromperie” nous amène à nous interroger sur la forte prévalence des MGF en Guinée, car nos données indiquent que les MGF pourraient être surdéclarées pour se conformer à la norme sociale apparente. Ce phénomène de “tromperie” a également été observé au Mali: l’examen physique effectué par des professionnels de la santé qualifiés a révélé que les organes génitaux de nombreuses filles déclarées excisées étaient en fait intacts; les mères de ces filles ont finalement admis avoir fait cette “fausse déclaration” pour éviter que leurs filles ne soient la cible de moqueries de la part de leurs paires, étant donné le conflit de valeurs sociales.^10^

De plus, le fait que nous n’ayons pas trouvé davantage de personnes ayant le profil de “courageuses” est. peut-être dû au fait que peu de Guinéens bénéficient des conditions de pouvoir qui leur permettent de concrétiser leur intention de ne pas pratiquer les MGF. Ce qui suit est. une discussion sur les profils et les sources de pouvoir spécifiques accessibles à nos participants selon leur typologie, qui leur permettent d’agir contre la norme en vigueur en matière de MGF.

Outre leur volonté de divulguer publiquement leur statut de non-pratique ou non, les profils des **“militants”** et des **“discrets”** sont très similaires, car ils n’éprouvent pas de problèmes avec la pression sociale, et montrent une accumulation de différentes formes de capital renforçant leur accès total au pouvoir. Ils démontrent généralement un capital culturel élevé via leur haut niveau d’éducation. Cela accroît par ricochet leurs chances d’accéder à un meilleur emploi (ex. dans l’ingénierie) et aux salaires élevés correspondants, comme en témoigne le capital économique élevé dont font preuve la plupart d’entre eux. Nous postulons que cela leur donne l’assurance d’avoir la capacité d’être financièrement indépendants de leur lignage si nécessaire, se sentant ainsi libres d’agir comme ils l’entendent. En outre, la plupart d’entre eux ont également tenu des propos démontrant une propension à l’individualisation, montrant une détermination à être indépendants de leurs réseaux sociaux et de leur lignage en cas de désaccord sur la question de la non-pratique des MGF. Mais surtout, ils ont tous mentionné faire partie d’un ou de plusieurs réseaux sociaux qui leur permettent de mettre en œuvre leur décision de ne pas faire exciser leurs filles. Ils vivent dans des niches sociales au sein desquelles les gens partagent des valeurs et des attitudes comparables relativement aux MGF, les épargnant ainsi d’être ostracisés ou stigmatisés.

Les **“courageuses”** présentent un capital total plus faible, c’est-à-dire un capital culturel faible/moyen et surtout un manque flagrant de capital social, étant donné la très forte pression sociale pour perpétuer la pratique des MGF qui prévaut en Guinée. Néanmoins, elles ont la capacité d’assumer leur décision de ne pas faire exciser leurs filles par le biais de leur capital économique, car leur indépendance économique relative leur donne le pouvoir de prendre le risque financier élevé d’agir en dépit qu’elles vivent dans un contexte d’interdépendance et qu’elles soient abandonnées par leur lignage. Cela suggère que le fait de bénéficier d’un seuil minimum de capital économique pourrait être l’une des conditions essentielles permettant aux Guinéens vivant à Conakry de mettre en œuvre leur décision, car cela leur donne les moyens individuels d’assurer leur subsistance et leur indépendance. Leur haut niveau de résilience leur permet également de faire valoir leur position malgré le lourd coût social qu’elles doivent supporter.

Les **“stratèges”** utilisent ce que certains pourraient appeler une “ruse” [31] pour éviter deux types d’écueils: 1) rester solidaires avec leur lignage et soumettre ainsi leurs filles à l’excision (ce qui n’est. pas une option pour elles), et 2) déclarer leur non-pratique et par conséquent être marginalisées, ostracisées et stigmatisées (ce qui équivaudrait à une “mort sociale”). Leur stratégie de dissimulation repose sur l’assurance que personne ne vérifiera physiquement si leurs filles ont réellement été excisées. Comme les “courageuses”, elles présentent un capital total plus faible, ayant un capital culturel intermédiaire ainsi qu’un capital social limité – avec certaines personnes avec qui elles sont étroitement liées qu’elles perçoivent comme des menaces pour l’intégrité physique de leurs filles. Elles “calculent” donc que le coût social – et très probablement financier – d’affirmer leur décision serait trop élevé, avec des conséquences (comme le divorce) qui seraient au-delà de ce qu’elles pourraient supporter. Malgré leur capital économique moyen à élevé, il n’est. pas clair, d’après leurs récits, si ces mères pourraient être indépendantes financièrement de leur mari et de leur famille.

Parallèlement, nous tenons à souligner que, selon leurs témoignages, une légère incision pratiquée dans le but de tromper l’apparence n’est. pas considérée par nos participantes comme étant une forme de MGF. Elles ont en effet affirmé qu’elles ne pratiquent pas les MGF et qu’elles ont trouvé des moyens pour protéger leurs filles contre cette tradition. Mais une telle incision – même si elle est. légère – est. par définition incluse dans la catégorie des MGF de type IV [1]. De plus, la pratique des MGF par des professionnels de la santé équivaut à la médicalisation des MGF, un phénomène qui est. en augmentation en Guinée [6, 9] bien qu’illégal [16, 17]. Par extension, il pourrait être interprété par certains que la médicalisation des MGF est. l’un des moyens que les “stratèges” utilisent pour éviter la pression sociale, mais d’après nos constats, nous ne le voyons pas ainsi. Nous soutenons cependant que cette méthode de “légère incision” utilisée pour protéger les filles contre une forme plus invasive de MGF ne devrait pas être encouragée.

En somme, ne pas vouloir que leurs filles soient excisées ne suffit pas. Afin de mettre en œuvre cette décision dans un contexte socioculturel de pratique quasi universelle des MGF, les parents doivent pouvoir s’appuyer sur des conditions d’*empowerment* significatives pour leur survie et leur bien-être, et nous postulons que ces principales sources de pouvoir sont les suivantes.

1. **Capital social favorable.** Nous sommes d’avis que puisque la mise en œuvre de la décision de ne pas faire subir de MGF aux filles est. avant tout une question sociale, la solution est. donc d’abord sociale. En effet, les parents qui décident d’abandonner la tradition doivent bénéficier d’un fort capital social qui soutient la non-pratique des MGF, ce qui implique d’avoir des points de vue et de faire des critiques similaires relativement à la tradition des MGF avec des personnes significatives de leur famille/réseau [46]. En outre, ce réseau doit inévitablement impliquer tant la mère que le père, et devrait de préférence inclure également la famille élargie – en particulier la grand-mère et les tantes paternelles. Ou encore, les familles qui veulent abandonner les MGF doivent vivre dans des environnements sociaux où les gens ne souhaitent pas savoir si l’excision est. pratiquée ou non, comme cela semble être de plus en plus le cas à Conakry.

2. **Capital économique suffisant permettant une indépendance financière.** En l’absence d’un capital social favorable, les parents doivent disposer d’un capital économique suffisant pour pouvoir agir individuellement et être financièrement indépendants si leur lignage leur retire son soutien financier. Notre échantillon de “déviants positifs” comprenait des personnes ayant un capital économique moyen à élevé, et aucune famille ayant un statut socio-économique faible. La Guinée étant connue pour être l’un des pays les plus défavorisés du monde [47], les individus en mode de survie peuvent avoir des options très limitées et donc se trouver dans une situation où ils sont incapables d’agir indépendamment de leurs réseaux de solidarité traditionnels [31]. Le capital économique pourrait également être un levier pour mettre en oeuvre la décision dans les cas où les parents de filles non-excisées soutiennent financièrement d’autres membres de la famille qui sont pro-MGF, en menaçant de couper ce soutien et en forçant donc ces derniers à respecter leur décision parentale [48].

Bien qu’il y ait peu d’études évaluant les répercussions économiques de la pratique des MGF sur les familles et les sociétés [49], il y a un manque flagrant de recherche concernant les implications économiques pour les familles qui ont choisi de ne pas pratiquer les MGF dans les pays à forte prévalence. Cependant, il est. connu que lorsque les individus les plus vulnérables augmentent leur pouvoir financier, ils peuvent se libérer des attentes normatives, car l’indépendance économique contribue à restructurer les relations de domination [42]. Il est. donc essentiel de travailler à la réduction des inégalités dans l’accès au capital social, culturel et économique pour parvenir à l’abandon des MGF en Guinée, en particulier pour les femmes. Cette recommandation est. conforme à la “théorie de la modernisation”, qui postule que l’augmentation des facteurs sociaux à l’échelle populationnelle tels que la scolarisation et l’emploi contribuera à l’abandon des MGF, très probablement à travers un processus d’individualisation [2, 50].

3. **Autres formes de résilience.** Les parents qui ne bénéficient pas d’un environnement social soutenant doivent compter sur d’autres sources de force pour faire face à la pression sociale, qui prend souvent la forme de la stigmatisation et de l’ostracisme. Cette “réponse positive à l’adversité” peut prendre différentes formes [32]. Certaines personnes peuvent trouver une certaine sérénité dans la pensée de protéger le bien-être de leurs filles, sachant que les MGF sont une pratique néfaste. D’autres peuvent s’appuyer sur leur foi, leur spiritualité ou leur pratique religieuse pour endurer ces problèmes. D’autres encore peuvent bénéficier du soutien de personnes individuelles, de réseaux sociaux ou de ressources communautaires.

4. **Protéger les filles contre le “vol”.** De nombreux parents doivent être constamment vigilants pour protéger leurs filles du risque d’être “volées” pour subir l’excision – un phénomène qui n’est. pas rapporté dans la littérature scientifique [51] mais qui est. bien connu des Guinéens. Dans une société collectiviste comme la Guinée, les enfants n’appartiennent pas aux parents, mais font partie d’un lignage au sein duquel les tantes et les grand-mères paternelles ont la responsabilité tacite de veiller au respect des normes et des valeurs [52]. Cette logique ne tient pas compte de la décision des parents, et ceux-ci doivent trouver des moyens de protéger leurs filles. Bien que certaines mères aient recours à la “tromperie” pour éviter ce genre de situation, nous soutenons que ce n’est. pas une solution viable, car elle repose sur un mensonge plutôt que sur un changement profond et durable de la mentalité de la société en ce qui concerne le maintien de l’intégrité physique des filles.

### Recommandations pour l’abandon des MGF en Guinée

Sur la base de nos résultats, nous suggérons les stratégies suivantes pour soutenir les parents à mettre en action, de manière durable, leur décision de ne pas faire subir de MGF à leurs filles.

1. **Augmenter le capital social des individus/familles.** Le renforcement des associations existantes ou le développement de nouvelles ressources communautaires dédiées à fournir aux personnes/familles qui ne pratiquent pas les MGF des sources de capital social devrait être une priorité, afin de soutenir ceux qui ne peuvent pas compter sur des réseaux familiaux ou communautaires pour les aider à mettre en oeuvre leur décision en toute sécurité et en toute liberté. Cette “restructuration” des systèmes de solidarité en Guinée [31] permettrait aux personnes/familles qui ne pratiquent pas les MGF de tisser des liens avec d’autres personnes qui partagent des points de vue et des valeurs communs, de leur fournir de nouvelles sources de soutien social [9], et ainsi de briser leur isolement et leur singularité. Et surtout, ces possibilités de capital social permettraient à d’autres personnes/familles de renforcer leur capacité d’action et leur *empowerment* [53, 54], ce qui leur permettrait de concrétiser leur intention de mettre fin à la pratique des MGF.

2. **Favoriser le capital économique des mères/familles.** Aider les gens à acquérir un pouvoir économique adéquat est. également une stratégie importante qui augmente la capacité des gens – en particulier des femmes – à assumer des décisions importantes [54, 55], comme abandonner les MGF. Les Guinéens qui doivent contourner les modes traditionnels de solidarité devraient avoir les moyens d’acquérir une indépendance financière si nécessaire. Cela pourrait se faire via 1) un soutien financier apporté par les réseaux associatifs [31]; 2) l’amélioration de l’accès à l’emploi; 3) sur le long terme, veiller à ce que la population atteigne un niveau d’éducation plus élevé, ce qui contribue par conséquent à l’accès à des emplois mieux rémunérés. Ceci est. d’autant plus urgent pour les femmes marginalisées et isolées qui sont seules responsables de leurs propres besoins et de ceux de leurs enfants, d’autant plus que la société guinéenne est. caractérisée par des discriminations et des injustices envers les femmes qui découlent de préjugés socioculturels [56]. Nous sommes donc d’avis que les ONG et les associations qui travaillent à promouvoir l’abandon des MGF, ainsi que celles qui travaillent à accroître le capital social et économique des Guinéens, devraient travailler ensemble et de manière concertée avec les ministères gouvernementaux concernés.

### Limites de l’étude

Bien que nous ayons cherché à inclure des personnes aux profils sociodémographiques variés, notre échantillon était finalement caractérisé par de nombreux participants très instruits et financièrement privilégiés, sans aucun participant ayant un faible niveau de capital économique. Il se peut que nous soyons passés à côté de certaines personnes moins éduquées et défavorisées en raison de notre technique de boule de neige et des contraintes de temps imposées par notre étude (recherche doctorale avec un financement limité). Par conséquent, nous n’avons pas pu recueillir les points de vue de certains “déviants positifs” qui auraient pu exister à Conakry. En outre, les sous-échantillons des “courageuses” et des “stratèges” ne représentaient qu’un faible pourcentage des participants de l’étude, mais présentaient néanmoins des profils et des expériences distinctifs. Une autre limite de notre étude est. que nous n’avons pas inclus le capital symbolique dans notre cadre conceptuel et dans notre guide d’entretien – lequel fait référence au prestige, à la réputation ou à la fierté des participants découlant de certaines réalisations ou d’un certain statut (ex. une profession hautement valorisée) [57]. Par conséquent, nous n’avons pas pu déterminer si cette forme de capital avait une incidence sur la capacité des participants à mettre en œuvre leurs décisions.

Malgré ces limites, une des forces de notre étude est. que la méthodologie de l’ethnographie focalisée a été une approche efficiente, efficace et innovante qui nous a permis de mieux comprendre dans quelles conditions les Guinéens vivant à Conakry mettent en œuvre leur décision de ne pas pratiquer les MGF. En outre, cette étude est. unique en fournissant une riche compréhension des expériences et des profils sociodémographiques de “déviants positifs” qui n’ont pas reçu toute l’attention nécessaire dans les études précédentes. Enfin, en mettant en avant les conditions nécessaires aux Guinéens pour mettre en oeuvre leur intention de ne pas faire exciser leurs filles, notre étude permet de produire des recommandations localement pertinentes pour les stratégies de santé publique.

### Perspectives pour la recherche future

Il est. crucial et urgent de reproduire cette étude dans les zones rurales et forestières de la Guinée, car les expériences vécues par les personnes qui ne pratiquent pas les MGF pourraient être différentes de celles qui ont été observées chez nos participants vivant dans un contexte urbain. Il faut également veiller à inclure davantage de personnes plus défavorisées et moins scolarisées, afin de mieux explorer quelles seraient les conditions optimales qui leur permettraient d’abandonner les MGF. De plus, bien que la stratégie de la “tromperie” semble être très bien connue à Conakry, elle est. virtuellement non documentée dans la littérature scientifique,^11^ et à peine discutée dans la littérature grise [48]: son ampleur est. par conséquent inconnue. Ce phénomène devrait être exploré plus avant dans de futures études. Enfin, notre étude n’a pas permis d’établir si le capital symbolique des participants est. impliqué dans leur capacité à mettre en œuvre leur décision, ce qui mériterait d’être exploré.

## Conclusions

Nos résultats suggèrent qu’il ne suffit pas de *vouloir* cesser de pratiquer les MGF. La principale condition pour que les parents *mettent en œuvre leur décision* dans le contexte socioculturel de Conakry est. de pouvoir compter sur un ou plusieurs réseaux de soutien social qui sont favorables à la non-pratique des MGF. Autrement, les mères et les familles doivent avoir la capacité d’agir individuellement et indépendamment de leur réseau de solidarité traditionnel, ce qui consiste essentiellement à être financièrement indépendants (capital économique suffisant). En somme, les mères/familles guinéennes vivant à Conakry doivent acquérir davantage de capital social et économique, et de façon intermédiaire davantage de capital culturel, afin de leur donner le pouvoir d’avoir du contrôle sur l’intégrité corporelle de leurs filles.

Cette étude était la première à explorer l’expérience de membres de familles qui ne pratiquent pas les MGF dans le contexte de fortes prévalence et pression sociale de la Guinée. Les résultats et les recommandations de cette recherche informeront les stratégies d’abandon des MGF et contribueront par conséquent à de meilleures interventions qui protégeront la santé et le bien-être des filles et des femmes.

### Remerciements

Nous tenons à exprimer notre sincère gratitude au Dr. Abdoul Habib Beavogui, directeur du *Centre national de formation et de recherche en santé rurale de Mafèrinyah* (Guinée), pour son soutien logistique et administratif, sa contribution à la réflexion sur les grandes lignes du projet, et ses encouragements tout au long de l’étude. Nous sommes également reconnaissants au professeur de sciences sociales Alpha Amadou Bano Barry du *Laboratoire d’analyse socio-anthropologique de Guinée (LASAG)* de l’Université générale Lansana Conté de Sonfonia (Guinée), pour son précieux soutien pour cette étude. Un grand merci au responsable du projet MGF du gouvernement guinéen (*Ministère de l’action sociale, de la promotion féminine et de l’enfance*), ainsi qu’aux associations communautaires guinéennes *Les Mêmes Droits pour Tous (MDT)*, le *Club des Jeunes Filles Leaders de Guinée*, la *Fondation Binta Ann pour les enfants et les femmes (FONBALE)* et *Sabou Guinée*, pour leur contribution indispensable au recrutement des participants à l’étude. Nos remerciements chaleureux à Dr. Nathalie Dinh (Université McGill, Canada) et Pr Frédéric Le Marcis (École normale supérieure de Lyon & Institut de recherche pour le développement, France) pour leurs précieux conseils pour le protocole de recherche; notre sincère gratitude au Pr Le Marcis d’avoir significativement inspiré l’analyse des données. Nous tenons également à remercier M. Saikou Oumar Sagnane, assistant de recherche guinéen en sciences sociales, qui a contribué avec diligence à la validation des traductions des langues locales vers le français. Et enfin, nous remercions infiniment nos participants pour leur enthousiasme et leur générosité dans la participation à notre étude.

### Contributions des auteurs

MHD, AD et DG ont dressé les grandes lignes du projet de recherche. MHD a élaboré le protocole de recherche sous la supervision de DG, lequel a ensuite été revu par AD. MHD et HM ont colligé et géré les données. MHD a effectué les analyses préliminaires, en tenant compte des commentaires de AD, HM et DG. MHD a rédigé cet article, en collaboration avec AD et DG. HM a révisé le manuscrit. Tous les auteurs ont revu et approuvé la version finale de l’article.

### Financement

MHD est. titulaire d’une bourse de formation doctorale du Fonds de recherche Québec – Santé (No. 257945), et détenait une “Bourse d’études supérieures du Canada Joseph-Armand-Bombardier” (Conseil de recherches en sciences humaines du Canada – CRSH, No. 767–2016-1530). Elle a également reçu une “*Alma Mater Fellowship*” de la Faculté de médecine et une bourse d’excellence d’entrée de l’Université McGill. Elle a reçu le soutien financier suivant pour ses voyages en Guinée: la “*Global Mental Health Travel Award”, des “Graduate Mobility Awards” et la “Norman Bethune Award for Global Health*” de l’Université McGill; et le Supplément pour études à l’étranger Michael-Smith du CRSH (No. 771–2017-0007). Elle a également reçu une subvention de dissémination des Instituts de recherche en santé du Canada / Institut des neurosciences, de la santé mentale et des toxicomanies pour cette étude (No. 410903). Les bailleurs de fonds n’ont joué aucun rôle dans la conception de l’étude, la collecte, l’analyse et l’interprétation des données, ni dans la rédaction du manuscrit.

### Disponibilité des données et du matériel

Les données générés et analysés au cours de la présente étude ne sont pas accessibles au public, afin de préserver l’anonymat des personnes interrogées. Toutefois, l’article présente suffisamment d’extraits verbaux anonymes pour illustrer les résultats.

### Approbation éthique et consentement à participer

Cette étude a reçu l’approbation éthique du Comité d’éthique de la recherche de l’Université McGill (No. A00-B39-18B) ainsi que du Comité national d’éthique pour la recherche en santé de la République de Guinée (No. 005/CNERS/18). Les chercheurs ont obtenu le consentement libre des participants après leur avoir expliqué et fait lire le formulaire de consentement détaillé. Les participants ont tous été invités à donner leur accord avant l’enregistrement des entretiens.

### Consentement à la publication

Non applicable.

### Intérêts concurrents

Nous n’avons pas d’intérêts concurrents à déclarer.

Reçu: 5 février 2020 Accepté: 23 avril 2020.

Publié en ligne: 6 mai 2020.

## Footnotes

1 La somme des ressources suivantes: radio; télévision ou ordinateur; téléphone (fixe ou mobile); réfrigérateur; cuisinière (électrique ou au gaz); table, chaises ou banc; armoire ou bibliothèque; matelas; électricité; eau courante; toilettes modernes; et sols finis (carrelage ou ciment). Un degré arbitraire a ensuite été attribué: un total de 10–12/12 a été considéré comme “élevé”, 6–9/12 comme “moyen”, et 0–5/12 comme “faible”.

2 Le nombre d’occupants divisé par le nombre de pièces. Un degré arbitraire a ensuite été attribué: ratio de 0–1 personne/pièce = “élevé”, 1–2 personnes/pièce = “moyen”, plus de 2 personnes/pièce = “faible”.

3 Le niveau économique global attribué à la famille était une “moyenne” de ces deux indicateurs (ex. élevé + élevé = élevé; moyen + faible = moyen).

4 Aucun *pattern* n’a été trouvé dans les récits concernant une relation possible entre les identités religieuses ou ethniques des personnes interrogées et la mise en œuvre de la décision de ne pas pratiquer les MGF.

5 Soutien social pour le pas pratiquer les MGF.

6 Les “stratèges” perçoivent que certaines personnes de leur entourage ne soutiennent pas la non-pratique des MGF.

7 Formation professionnelle: ex. formation de sage-femme, certifications techniques.

8 Le récit n’a pas permis de classer ces participants dans l’un ou l’autre.

9 Ils ont utilisé le verbe français “voler”, qui pourrait être défini dans ce contexte comme un “vol” temporaire pour le seul but de soumettre la fille à l’excision pour respecter les normes socioculturelles.

10 Pr Touré M: Communication personnelle (2019). Ce constat n’est. pas officiellement publié, car il a été découvert par hasard.

11 Barry a fait référence aux familles qui “font semblant” de faire exciser leurs filles, mais il n’a pas décrit ce phénomène plus en détails [9].

## References

[1] World Health Organization (2020). Female genital mutilation (fact sheet).

[2] Andro A, Lesclingand M (2016). Female genital mutilation. Overview and current knowledge. Institut National d’Études Démographiques | “Population”.

[3] Institut National de la Statistique (INS) et ICF (2018). Enquête démographique et de santé en Guinée 2018: Indicateurs clés Conakry, Guinée, et Rockville, Maryland, États-Unis d’Amérique.

[4] Mulongo P, Hollins Martin C, McAndrew S (2014). The psychological impact of Female Genital Mutilation/Cutting (FGM/C) on girls/women’s mental health: a narrative literature review. J Reprod Infant Psychol.

[5] UNICEF (2016). Female genital mutilation/cutting: A global concern.

[6] UNICEF (2013). Female Genital Mutilation/Cutting: A statistical overview and exploration of the dynamics of change. New York, United States of America.

[7] African Commission on Human and Peoples’ Rights (2003). Protocol to the African charter on human and peoples’ rights on the rights of women in Africa.

[8] Organisation of African Unity (1990). African charter on the rights and welfare of the child. Addis-Abeba, Ethiopia.

[9] Barry AAB (2015). La perpétuation des MGF en Guinée – Analyse socio-anthropologique des déterminants.

[10] Doucet MH, Pallitto C, Groleau D (2017). Understanding the motivations of health-care providers in performing female genital mutilation: an integrative review of the literature. BMC Reprod Health.

[11] Marsh DR, Schroeder DG, Dearden KA, Sternin J, Sternin M (2004). The power of positive deviance. Br Med J.

[12] Njue C, Askew I (2004). Medicalization of Female Genital Cutting Among the Abagusii in Nyanza Province, Kenya. Frontiers in Reproductive Health Program.

[13] Nutrition Working Group CSCaRGC. Positive Deviance / Hearth: A Resource Guide for Sustainably Rehabilitating Malnourished Children. Washington, D.C, United States of America; 2002.

[14] Population Council (2008). Female genital mutilation abandonment program – Evaluation summary report.

[15] Institut National de la Statistique (INS) et ICF (2019). Enquête démographique et de santé en Guinée 2018. Edited by ICF Ie. Conakry, Guinée, et Rockville, Maryland, United States of America.

[16] Barry AAB (2017). Guinée: l’impact des stratégies de promotion de l’abandon des mutilations génitales féminines.

[17] Gouvernement de la République de Guinée (2008). Code de l’enfant guinéen (LOI L/2008/011/AN du 19 août 2008) – Section VII: Des violences exercées à l’encontre des enfants – Article 405 – Les mutilations génitales féminines.

[18] Gouvernement de la République de Guinée – Ministère de la Justice (2016). Nouveau code pénal – Section II: Des mutilations génitales féminines (Articles 258–261).

[19] Barry AAB (2019). Étude sur la perception des bénéfices que les femmes et les communautés trouvent dans la pratique des MGF.

[20] Bourdieu P (1984). Distinction: A social critique of the judgement of taste.

[21] Yoder PS, Mahy M (2001). Female genital cutting in Guinea: Qualitative and quantitative research strategies. DHS Analytical Studies No 5.

[22] Knoblauch H. Focused Ethnography. Forum: Qualitative Social Research. 2005;6(3).

[23] Triandis HC (2001). Individualism-collectivism and personality. J Pers.

[24] Van Bavel H, Coene G, Leye E (2017). Changing practices and shifting meanings of female genital cutting among the Maasai of Arusha and Manyara regions of Tanzania. Cult Health Sex.

[25] Green J, Thorogood N (2014). Qualitative Methods for Health Research.

[26] Higginbottom GMA, Pillay JJ, Boadu NY (2013). Guidance on performing focused ethnographies with an emphasis on healthcare research. Qual Rep.

[27] Miles MB, Huberman AM, Saldana J (2014). Qualitative data analysis: A methods sourcebook.

[28] Maxwell JA (2010). Using Numbers in Qualitative Research. Qualitative Inquiry.

[29] Bourdieu P (1986). The forms of capital. In: Handbook of theory and research for the sociology of education. Edited by J. Richardson. New York, Greenwood.

[30] Bourdieu P (1979). Les trois états du capital culturel. Actes de la recherche en sciences sociales.

[31] Vuarin R, Leimdorfer F, Werner JF, Gérard E, Tiékoura O (1997). L’Afrique des individus. Itinéraires citadins dans l’Afrique contemporaine (Abidjan, Bamako, Dakar, Niamey), Édition Karthala. Paris, France.

[32] Kirmayer LJ, Sehdev M, Whitley R, Dandeneau SF, Isaac C. Community resilience: Models, metaphors and measures. Journal de la santé autochtone. 2009:62–117.

[33] Kirchherr J, Charles K (2018). Enhancing the sample diversity of snowball samples: Recommendations from a research project on anti-dam movements in Southeast Asia. PLoS One.

[34] Gouvernement de la République de Guinée. Code de l’enfant guinéen (LOI L/2008/011/AN du 19 août 2008). Conakry, Guinée; 2008.

[35] Sutton J, Austin Z (2015). Qualitative Research: Data Collection, Analysis, and Management. Can J Hosp Pharm.

[36] Ritchie J, Spencer L (1994). Qualitative data analysis for applied policy research. Analyzing qualitative data. Edited by A. Bryman and R.G. Burgess.

[37] Seale C, Silverman D (1997). Ensuring rigour in qualitative research. Eur J Pub Health.

[38] Whittemore R, Chase SK, Mandle CL (2001). Validity in Qualitative Research. Qual Health Res.

[39] O’Brien BC, Harris IB, Beckman TJ, Reed DA, Cook DA (2014). Standards for reporting qualitative research: a synthesis of recommendations. Acad Med.

[40] Triandis HC (1989). The self and social behavior in differing cultural contexts. Psychol Rev.

[41] Triandis HC, Gelfand MJ (1998). Converging measurement of horizontal and vertical individualism and collectivism. J Pers Soc Psychol.

[42] Bouju J, De Bruijn M (2008). Violences structurelles et violences systémiques. La violence ordinaire des rapports sociaux en Afrique. Bulletin de l’APAD.

[43] Mackie G (1996). Ending Footbinding and Infibulation: A Convention Account. Am Sociol Rev.

[44] Mackie G (2000). Female genital cutting: the beginning of the end. In: Female “circumcision” in Africa: culture, controversy, and change. Edited by Shell-Duncan B, Hernlund, Y.: Lynne Rienner.

[45] Shell-Duncan B, Wander K, Hernlund Y, Moreau A (2011). Dynamics of change in the practice of female genital cutting in Senegambia: testing predictions of social convention theory. Soc Sci Med.

[46] Van Bavel H. At the intersection of place, gender, and ethnicity: changes in female circumcision among Kenyan Maasai. Gend Place Cult. 2019:1–22.

[47] Central Intelligence Agency (CIA) (2019). The World FactBook – Africa: Guinea.

[48] Dembélé M, Oertli A, Woehrel A (2018). Rapport de mission en Guinée du 7 au 18 novembre 2017. Office français de protection des réfugiés et apatrides (OFPRA) avec la participation de la Cour nationale du droit d’asile (CNDA); France.

[49] Mpinga EK, Macias A, Hasselgard-Rowe J, Kandala N-B, Félicien TK, Verloo H, Bukonda NKZ, Chastonay P (2016). Female genital mutilation: a systematic review of research on its economic and social impacts across four decades. Glob Health Action.

[50] Boyle E (2002). Female genital cutting: cultural conflict in the global community.

[51] Shell-Duncan B, Hernlund Y (2006). Are there “stages of change” in the practice of female genital cutting?: Qualitative research findings from Senegal and The Gambia. Afr J Reprod Health.

[52] Fassin D (2005). 1. L’ordre moral du monde – Essai d’anthropologie de l’intolérable. Les constructions de l’intolérable. Études d’anthropologie et d’histoire sur les frontières de l’espace moral. La Découverte | « Recherches ». Edited by P. Bourdelais Patrice & D. Fassin. Paris, France.

[53] Mayoux L (2001). Tackling the Down Side: Social Capital, Women’s Empowerment and Micro-Finance in Cameroon. Dev Chang.

[54] Nega F, Mathijs E, Deckers J, Tollens E (2009). Gender, social capital and empowerment in northern Ethiopia. Munich Personal RePEc Archive.

[55] World Bank (2019). Guinea: The economic benefits of a gender inclusive society. Washington, DC, United States of America.

[56] Haut-Commissariat des Nations Unies aux droits de l’homme (2016). Rapport sur les droits humains et la pratique des mutilations génitales féminines/excision en Guinée.

[57] Bourdieu P (1987). Choses dites.

